# Silver-Bacterial Cellulose-*Areca catechu* Extract Nanocomposite Biofilm for Sustainable Food Packaging: Synthesis, Characterization, and Antibacterial Activity

**DOI:** 10.3390/molecules31193373

**Published:** 2026-09-23

**Authors:** Ririn Nur Alfiani, Anastasia Wheni Indrianingsih, Fransiska Sri Herwahyu Krismastuti, Anjar Windarsih, Eka Noviana, Eka Rahayu, Tri Wiyono

**Affiliations:** 1 Research Center for Food Technology and Processing, National Research and Innovation Agency (BRIN), Yogyakarta 55861, Indonesia; riri013@brin.go.id (R.N.A.); anja003@brin.go.id (A.W.); ekar004@brin.go.id (E.R.); triw013@brin.go.id (T.W.); 2 Research Center for Molecular Chemistry, National Research and Innovation Agency (BRIN), Tangerang Selatan 15314, Indonesia; fran005@brin.go.id; 3 Department of Pharmaceutical Chemistry, Faculty of Pharmacy, Universitas Gadjah Mada, Yogyakarta 55281, Indonesia; eka.noviana@ugm.ac.id

**Keywords:** biofilm, bacterial cellulose, *Areca catechu*, antibacterial activity, sustainable food packaging

## Abstract

Bacterial cellulose (BC) is a low-cost, biodegradable biopolymer with excellent mechanical strength and water-holding capacity. It is an attractive material for sustainable food packaging; however, native BC lacks intrinsic antibacterial activity. In this study, silver-bacterial cellulose-*Areca catechu* nanocomposite biofilms were synthesized through in situ reduction of Ag^+^ on BC films, followed by impregnation with *A. catechu* fruit extract at concentrations of 0–30 mg/mL. The resulting films were characterized by scanning electron microscopy-energy-dispersive X-ray spectroscopy (SEM-EDX), Fourier-transform infrared (FTIR) spectroscopy, X-ray diffraction (XRD), swelling capacity, and color analysis. The films were evaluated for antibacterial activity against *Escherichia coli* and *Staphylococcus aureus* using the disk-diffusion method. SEM-EDX confirmed the successful deposition of silver nanoparticles within the BC matrix, while FTIR and XRD indicated changes in functional groups and crystallinity following film modification. The swelling capacity of the films ranged from approximately 145 to 370%, with the film containing 20 mg/mL extract exhibiting the highest capacity. The film containing 10 mg/mL extract exhibited the strongest antibacterial activity, with inhibition zones of 6.50 mm against both *S. aureus* and *E. coli*, outperforming the pure BC and Ag-BC films as well as the higher-concentration formulations. Liquid chromatography-high-resolution mass spectrometry (LC-HRMS) profiling of the *A. catechu* extract putatively identified more than 120 metabolites dominated by alkaloids such as arecoline and guvacoline. Other detected metabolites include catechin, protocatechuic acid, penicillic acid, nootkatone, and phytosphingosine, which are known to have antibacterial activity. Taken together, these findings demonstrate that the developed nanocomposite biofilms are promising candidates, low-cost, and environmentally friendly antibacterial materials with potential for further development as active food packaging materials. Further safety and migration assessments are required to establish their suitability for direct food-contact applications.

## 1. Introduction

The accumulation of petroleum-based plastic packaging waste has become a pressing environmental concern [1]. The plastic packaging materials are difficult to decompose and can persist in the environment, where they may gradually fragment into microplastics that pose potential risks to human health [2,3]. This problem has driven increasing interest in biodegradable and renewable materials for the food industry [4]. Among biopolymers explored for this purpose, bacterial cellulose (BC) has attracted particular attention because of its high purity, mechanical strength, water-holding capacity, and biodegradability [5,6]. BC can be produced from agricultural by-products such as coconut water waste, offering additional environmental and economic advantages [6]. Despite these favorable properties, native BC lacks intrinsic antimicrobial activity [7]. This limitation restricts its direct application as an active packaging material capable of inhibiting the growth of food-spoilage and foodborne pathogenic microorganisms.

To impart antimicrobial functionality, silver nanoparticles (AgNPs) are widely incorporated into biopolymer matrices, including BC [8,9]. AgNPs have broad-spectrum activity against Gram-positive and Gram-negative bacteria [9,10]. Ag-BC composite films have therefore been explored as active food packaging materials that combine the mechanical and barrier properties of BC with the antibacterial action of silver [8,11]. However, relying solely on AgNPs raises concerns regarding cost and the risk of bacterial resistance. This issue has driven the search for complementary, plant-derived antibacterial agents that can be combined with Ag-BC systems to enhance their bioactivity. For example, Ag-BC composites have been combined with *Clitoria ternatea*, *Moringa oleifera*, and *Astrocaryum aculeatum* to create functional food packaging with antimicrobial activity against bacteria such as *Escherichia coli*, *Staphylococcus aureus*, and *Pseudomonas aeruginosa* [12,13].

*Areca catechu* L. (also known as areca nut or betel nut) is an economically important crop widely cultivated across Indonesia and other tropical regions. Its fruit is rich in bioactive compounds, including alkaloids such as arecoline, arecaidine, guvacoline, and guvacine, as well as tannins, flavonoids, and other phenolic compounds [14]. These compounds have been reported to possess antibacterial, antioxidant, and anti-inflammatory properties [14,15,16]. A previous study showed that *A. catechu* methanol extract and its associated fractions exhibited minimum inhibitory concentrations (MICs) of 62.5–250 µg/mL against several strains of *S. aureus*, *S. mutans*, and *M. spegmatis* [17]. Another study reported that ethanol and water extracts of *A. catechu* were active against *B. subtilis* and *S. aureus*, with MIC values ranging from 0.094 to 1.56 mg/mL [18]. Despite its potential, *A. catechu* fruit remains an underutilized resource, and its valorization as a functional ingredient in active packaging materials has not been widely explored. Combining AgNPs with BC and *A. catechu* extract offers an opportunity to develop a multifunctional nanocomposite film, in which the plant extract may act synergistically with silver to enhance antibacterial performance.

In this study, silver-bacterial cellulose-*A. catechu* extract nanocomposite biofilms were synthesized at extract concentrations of 0–30 mg/mL and characterized in terms of their morphology, elemental composition, functional groups, crystallinity, swelling capacity, and color properties. The antibacterial activity of the resulting films was evaluated against the food-relevant pathogens, including *E. coli* and *S. aureus*. The phytochemical composition of the *A. catechu* extract was profiled by LC-HRMS to identify the bioactive constituents likely responsible for the antibacterial activity. Overall, this study aims to provide a scientific basis for the development of Ag-BC-*A. catechu* nanocomposite biofilms as a sustainable, antibacterial packaging material that valorizes coconut water waste and underutilized *A. catechu* fruit.

## 2. Results and Discussion

### 2.1. Morphology and EDX Measurement

The surface morphology of pure bacterial cellulose (BC), Ag-BC prepared without *A. catechu* extract, and Ag-BC synthesized with different concentrations of *A. catechu* extract were observed under SEM (Figure 1), while the elemental composition was confirmed by EDX analysis (Figure S1 and Table 1).

The pure BC, as shown in Figure 1a, has a characteristic three-dimensional interconnected nanofibrous network with randomly oriented cellulose fibrils and intrinsic pore architecture. The width of the nanofibrils in BC was around 26–50 nm. Ag-BC without *A. catechu* extract displays a different surface morphology (Figure 1b). The characteristic nanofibrous network of BC was almost completely covered by deposition of silver. The Ag^+^ ions were rapidly reduced by sodium citrate. Due to the absence of *A. catechu* extract, growth of Ag was uncontrollable, causing severe aggregation of Ag onto the BC fibers and masking the fibrillar structure of BC. The Ag nanoparticles exhibited an estimated particle size of approximately 100–125 nm (Figure 1b) and 90–100 nm (Figure 1c–e) based on SEM image analysis; however, particle aggregation in some regions limited the accurate determination of individual nanoparticle dimensions.

After the addition of *A. catechu* extract (Figure 1c–e), the surface morphology of the Ag-BC was changed. The nanofibrous network became visible again and could be differentiated from the presence of silver. This may indicate the role of phytochemicals in the extract as a capping and stabilizing agent in controlling the growth of silver particles and reducing aggregation. Consequently, the Ag was uniformly distributed on the surface of BC; thus, the fibrillar structure of BC could be observed by SEM. However, increasing the concentration of the extract changed the morphology of Ag-BC since the nanofibrous structure became invisible again (Figure 1e). This may be due to the excessive extract concentration covering the nanofibrous network of BC. This demonstrates the concentration dependence of *A. catechu* extract in the formation of silver onto the BC fibers.

In the pure BC, the peak of Ag was not detected, confirming the absence of silver in the sample (Figure S1a, Table 2). After the addition of Ag without *A. catechu* extract, the peak corresponding to Ag appeared in the EDX spectrum (Figure S1b) with the element percentage of 0.6 (Table 2), confirming the rapid deposition of silver onto BC, as shown in the SEM image. The addition of a low-concentration extract (10 mg/mL) reduced the percentage of silver deposited onto BC (Table 2). As the concentration of *A. catechu* extract increased from 10 mg/mL to 20 mg/mL, the Ag content determined by EDX increased correspondingly (from 0.1% to 0.3%), which can also be observed by SEM: the higher the extract concentration, the more silver on the BC. However, further increasing the extract concentration to 30 mg/mL, the Ag content remained the same (0.3%), indicating that the silver deposition had reached a saturation level. This means that the available Ag^+^ ions had been efficiently reduced at an extract concentration of 20 mg/mL; adding more extract did not increase the amount of deposited silver. Despite the similar Ag content, the SEM image of the highest extract concentration (30 mg/mL) showed that the nanofibrous structure became invisible (Figure 1e). Therefore, the layer deposited on the BC was not attributed to additional Ag deposition but rather to the formation of a thin bioorganic layer due to the excess of phytochemicals on the BC surface that act predominantly as capping agents instead of promoting nanoparticle formation. The map of silver distribution in the composite film of Ag-BC-*A. catechu* was presented in Figure S2.

### 2.2. FTIR Study

The characteristic vibrational bands in *A. catechu* extract were demonstrated through the FTIR spectrum (Figure 2), indicating a rich phytochemical profile comprising polyphenols and flavonoids. A broad absorption peak observed at 3268 cm^−1^ corresponded to the vibration of -OH stretching from hydroxyl groups in polyphenols and water molecules [19]. The peaks at 2916 cm^−1^ and 2846 cm^−1^ were assigned to the stretching vibrations of -CH, -CH_2_, and -CH_3_ aliphatic groups, comprising asymmetric and symmetric vibrations, respectively. Meanwhile, a distinct peak at 1700 cm^−1^ was associated with the stretching vibration of carbonyl (C=O) or ester groups in phenolic derivatives [20]. In addition, the band observed at 1600 cm^−1^ was attributed to the stretching vibration of the C=C aromatic ring, typical of phenolic compounds. The peaks consisting of 1463, 1436, 1369, and 1269 cm^−1^ highlighted the bending vibration of -CH, -CH_2_, and -CH_3_. Furthermore, the stretching vibration of C-O-C and C-O was observed at the bands of 1155, 1103, and 1058 cm^−1^ [21].

For pure bacterial cellulose (Figure 2(A)), the characteristic cellulose fingerprint peaks were observed, including the O–H stretching at 3268 cm^−1^, aliphatic C–H stretching at 2916 cm^−1^, C=C aromatic ring stretching vibration around 1600 cm^−1^, and C–O–C glycosidic linkage and C–O stretching modes at 1155, 1103, and 1058 cm^−1^. In the spectrum of Ag-BC without extract (Figure 2(B)), the core framework of the bacterial cellulose network remained largely intact. However, subtle alterations in the intensity and minor shifts in the hydroxyl region (3268 cm^−1^) indicated weak non-covalent or electrostatic interactions between the silver nanoparticles (AgNP) and the hydroxyl oxygen atoms of the cellulose matrix [22]. Upon incorporation of *A. catechu* extract into the composite films (Figure 2(C–E)), corresponding to 10, 20, and 30 mg/mL extract concentrations, respectively, the main characteristic peaks of bacterial cellulose were preserved, confirming that the structural integrity of the cellulose matrix remained uncompromised. As the concentration of *A. catechu* extract increased, the relative intensities of the bands corresponding to polyphenol-associated functional groups, particularly the O–H stretching (3268 cm^−1^) and C=C aromatic ring stretching (1600 cm^−1^) regions, showed concentration-dependent variations and peak broadening. In addition, the peak at 1700 cm^−1^ did not appear in all samples (Figure 2(A–E)), except in *A. catechu* extract. These spectral changes confirm hydrogen bonding interactions and coordination linkages between the phytochemical compounds of *A. catechu*, the silver nanoparticles, and the bacterial cellulose matrix. Following the synthesis of the Ag-BC nanocomposites, the films were repeatedly washed using deionized water. This step can effectively remove most unreacted, residual sodium citrate and soluble citrate oxidation byproducts. Any remaining traces of sodium citrate or its oxidation products exhibited characteristic asymmetric and symmetric carboxylate stretching bands, which might appear around 1600–1580 cm^−1^, along with the carbonyl stretching around 1700 cm^−1^. In this study, these signals overlapped with the prominent polyphenolic C=O and C=C ring stretching modes of *A. catechu* extract and the adsorbed water/hydroxyl bending modes of bacterial cellulose.

### 2.3. XRD Study

The XRD patterns of pure and composite BC are shown in Figure 3. A cellulose-related diffraction peak was observed at approximately 2θ = 21–22°, whereas the reflections at 42.71°, 63.25°, and 80.72° were already present in pure BC and cannot be specifically attributed to metallic Ag. In contrast to previous reports on Ag/BC composites showing characteristic face-centered cubic Ag reflections at approximately 38.1°, 44.2°, 64.4°, and 76.7° [23], these reflections were not clearly detected in the present Ag-containing films. This may be associated with the relatively low Ag content or insufficiently crystalline Ag domains to produce distinguishable diffraction peaks. Therefore, XRD alone does not provide conclusive evidence for crystalline metallic Ag in the films. The presence and spatial distribution of Ag were independently supported by EDX spectra and EDS elemental mapping (Figures S1 and S2).

The XRD analysis further revealed differences in the structural ordering of cellulose among the treatments. The crystallinity index (CrI) was 51.26% for pure BC and 80.93% for Ag-treated BC, while addition of 10 mg/mL *A. catechu* extract resulted in a comparable CrI of 81.31%. Increasing the extract concentration to 20 mg/mL markedly decreased the CrI to 28.38%. In contrast, the crystallinity index after treatment using the extract concentration of 30 mg/mL could not be reliably determined because the cellulose-related reflection was insufficiently detected. The corresponding apparent crystallite dimensions estimated using the Scherrer equation were 5.25, 5.68, 5.77, and 6.31 nm, respectively (Table S1). Nevertheless, the higher CrI observed after Ag treatment should not be interpreted as direct evidence that Ag promoted cellulose crystallization, particularly because Ag was introduced after BC film formation. Conversely, these values represent comparative changes in cellulose structural ordering among the treated films. Such caution is particularly important for bacterial cellulose because XRD-derived crystallinity indices can be affected by peak overlap, crystallite size, background treatment, and preferred orientation. Consequently, empirical CrI values are more appropriate for evaluating relative structural changes within similarly analyzed samples rather than absolute crystalline fractions [24,25].

Although increased cellulose crystallinity is generally associated with reduced water accessibility and swelling, the swelling behavior of BC-based composites is not governed solely by crystallinity. Other structural and physicochemical factors, including the porous nanofibrillar networks, availability of hydrophilic functional groups, and interactions among composite constituents, can substantially influence water uptake and retention [6,26]. Therefore, the swelling behavior observed in the present Ag-BC-*A. catechu* films should not be interpreted solely on the basis of their Segal crystallinity index.

### 2.4. Swelling Capacity

Swelling capacity refers to the ability of nanocomposite biofilms to absorb water as food packaging materials [27]. Swelling tests revealed marked differences among the five nanocomposite biofilm formulations (Figure 4). The pure bacterial cellulose/BC (without Ag and *A. catechu*) exhibited a moderate swelling ratio of ~234%, adsorbing about 2.3 times its own weight in water. Incorporating Ag into BC could enhance the swelling capacity to around 350%. Furthermore, incorporating *A. catechu* into Ag-BC could decrease or increase swelling capacity at different concentrations. Ag-BC-*A. catechu* 2 film had a higher swelling capacity compared to others, with a value of 370%. The increased swelling capacity upon the addition of Ag may be due to its porous and hydrophilic properties [28,29]. Furthermore, films C and E, with the addition of Ag and *A. catechu* (concentrations of 10 mg/mL and 20 mg/mL), may reduce swelling capacity. Several studies also reported that the addition of AgNPs could reduce the swelling degree of the synthesized film, for example, in the nanocomposite based on chitosan/Ag NPs [30] and the chitosan/pectin/ZnO porous film [31]. Swelling capacity often relates to the material’s ability to absorb moisture, which can help protect food from excess humidity and maintain quality [32,33]. However, excessive swelling may compromise mechanical integrity [34]. High water uptake often leads to plasticization and weakening of the film structure, resulting in reduced mechanical strength and increased risk of deformation or failure. Studies on hydrophilic films show that moisture content significantly affects their mechanical properties, typically reducing strength and elasticity as water uptake increases [35,36]. Therefore, the biofilm has the potential to be used for food packaging with limited water exposure. Meanwhile, its application to foods with high moisture content for long periods of time requires further evaluation of its mechanical stability and barrier properties under wet or humid conditions.

### 2.5. Color Measurement

The appearance and color of food packaging may influence consumer preferences. L*, a*, and b* were used as color parameters for films. L* represents lightness and darkness, a* represents greenness and redness, and b* represents blueness and yellowness [37]. The color analysis of the films was shown in Table 2. The study revealed that incorporating *A. catechu* into the film formulation decreased brightness (L*) while increasing redness (a*) and yellowness (b*). There were significant differences (*p* < 0.05) in the L*, a*, and b* values among the films. The L* value of film BC is 43.25 ± 0.34, the highest among all films. The addition of Ag and *A. catechu* decreased the L* value, indicating an increase in darkness. The L* values of the Ag-BC, Ag-BC-*A. catechu* 1, Ag-BC-*A. catechu* 2, and Ag-BC-*A. catechu* 3 films are 30.80 ± 0.10, 35.16 ± 0.22, 31.50 ± 0.33, and 35.42 ± 0.18, respectively. The shift from light to dark in the films is due to the phenolic compounds present in *A. catechu*, which improve the scattering and reflection of light from the films [38]. The a* value trends upward from BC to BC-Ag-*A. catechu* 3, with values ranging from −0.54 ± 0.03 to 6.42 ± 0.90. This indicates an increase in redness. Furthermore, increasing the *A. catechu* concentration decreases the b* value from 12.72 ± 0.55 to 10.48 ± 0.38.

The color of *A. catechu* powder can be described as yellowish or reddish-brown [39]. Klaichoi et al. [40] reported that *A. catechu* produces shades ranging from brown to reddish-brown; thus, the color may be influenced by phenolic content in *A. catechu*. According to Stohs et al. [41] catechin and epicatechin are major contributors in *A. catechu* extracts, which has been proven in both in vitro and in vivo studies. Therefore, the phenolic contents in *A. catechu* were suggested to influence the brightness, redness, and yellowness level of films [42]. Previous research reported that *A. catechu* contains a high amount of catechin with a value of 13–33% [43].

### 2.6. Antibacterial Activity

The disk diffusion method is a widely used laboratory procedure to assess the antibacterial activity of substances and determine the susceptibility of bacteria to antibiotics [44]. The diameter of the clear zone around each disk is measured, usually in millimeters, using a caliper. The advantages of this method are that it is simple, cost-effective, reproducible, and widely accepted for routine testing [45]. A larger zone indicates greater antibacterial activity of the tested substance. The antibacterial activity of Ag-BC-*A. catechu* films was presented in Table 3.

The results of the antibacterial test with *S. aureus* showed that film C had the highest inhibition value of 6.50 mm, followed by films B, E, and D. The reported values were only the inhibition area beyond the 10 mm test square film. Amoxicillin as a positive standard had a very high inhibition value against *S. aureus* of 16.75 mm. Meanwhile, film C also had the highest activity against *E. coli* with a value of 6.50 mm, followed by films E, B, and D. Amoxycillin as a positive standard had a very high inhibition value against *E. coli* of 16.50 mm. The representative photographs of the antibacterial plates were presented in Figure S3. Silver nanoparticles exhibit strong antibacterial activity against both *S. aureus* (Gram-positive) and *E. coli* (Gram-negative) through multiple, simultaneous mechanisms [46]. AgNPs interact with and damage bacterial cell membranes, causing leakage of cellular contents and loss of membrane integrity. AgNPs also induce the formation of reactive oxygen species (ROS), which damage proteins, lipids, and DNA [47]. AgNPs cause protein leakage and DNA condensation, impairing replication and cellular processes [48]. Disk diffusion assays, commonly used to demonstrate antimicrobial properties, rely on the diffusion of silver ions through the medium, which is facilitated by the high surface area of nanoparticles but not by the migration of the nanoparticles themselves [49]. The antimicrobial effect observed in these assays is substantially due to the release and diffusion of Ag^+^ ions from the material into the agar, rather than the physical movement of nanoparticles [50]. The limitation of this study is that if these compounds are not readily released from insoluble BC-based films or do not diffuse efficiently, the observed inhibition zones may not accurately reflect antimicrobial activity [51].

On the other hand, extracts from *A. catechu* show antibacterial effects against a variety of pathogenic bacteria, including both Gram-positive and Gram-negative species. Methanol, ethanol, and aqueous extracts have all demonstrated inhibitory effects, with methanol and ethanol extracts often showing stronger activity than water extracts [52,53]. Methanol and ethanol extracts generally exhibit higher antibacterial activity compared to aqueous extracts, likely due to better extraction of bioactive compounds such as alkaloids, tannins, and polyphenols [52,53]. The antibacterial properties of *A. catechu* extracts are attributed to the presence of flavonoids, tannins, saponins, and alkaloids in the fruit, which can disrupt bacterial cell walls and inhibit growth [16,54].

### 2.7. LC-HRMS Analysis of A. catechu Extract

As shown in Table S2, the results of LC-HRMS analysis revealed that *A. catechu* fruit extract contains various phytochemicals, primarily alkaloids and polyphenolic derivatives. The major putatively identified compounds (Table 4) measured include alkaloid compounds, namely arecoline (33.5%), nicotinyl alcohol (10.3%), guvacoline (5.8%), caffeine (3.2%), betaine (2.9%), Methyl isonicotinate, and Tranexamic acid. These results were supported by Zhou et al. [55] which states that arecoline biosynthesis involves pyridine-derived intermediates. The second major group is fat (and fat-soluble compounds), including 9-Oxo-10(E),12(E)-octadecadienoic acid; 2-[(5Z)-5-tetradecenyl]cyclobutanone; 9,10-Dihydroxystearic acid; 12,13-DiHOME; Stearamide, Ethyl palmitoleate, Phytosphingosine, Nootkatone, and α-Eleostearic acid. In addition, phenolic and flavonoid compounds include such as Cyanidanol and protocatechuic acid. Additionally, analytical evidence is provided in the form of fragmentation profiles of selected compounds from the *A. catechu* extract chromatogram, which can be seen in Table S3.

These molecules have been reported to possess antimicrobial activity. Caffeine inhibits bacteria by inhibiting RNA synthesis in phytopathogenic bacteria, while also altering cell morphology and inhibiting cell division [10]. Cyanidanol is said to interact with cell membranes, penetrating phospholipids and causing permeability disruption [56]. In cancer cells, cyanidanol induces cell apoptosis through mitochondrial modulation, such as increasing Bax and decreasing Bcl-2, leading to caspase activation and cell cycle arrest. Meanwhile, Benjumea et al. [57] reported that tranexamic acid (TXA) has a synergistic effect with antibiotics such as vancomycin and gentamicin, thought to be due to its ability to disrupt biofilm formation by inhibiting bacterial aggregation, thus facilitating antibiotic access [57].

Phytosphingosine and nootkatone compounds act directly on bacterial cell structures, according to several studies. Phytosphingosine works by damaging the cytoplasmic membrane, which causes cell lysis [58]. Its activity is very rapid, capable of killing bacteria within hours. Meanwhile, nootkatone, a sesquiterpene, has bacteriostatic and bactericidal activity against Gram-positive bacilli such as Listeria monocytogenes and Corynebacterium diphtheriae [59]. Nootkatone effectively inhibits biofilm formation in *S. aureus* by suppressing the expression of genes involved in biofilm formation, such as sarA, icaA, and agrA [60]. Another potent compound is Protocatechuic acid (PCA). In bacteria, PCA disrupts cell membrane integrity, causing membrane depolarization, decreased intracellular pH (pHin), and leakage of ATP and potassium ions (K+) [61]. In cancer cells, PCA induces apoptosis through activation of the JNK/p38 MAPK pathway, increased Bax expression, and decreased Bcl-2, suggesting a dual role that depends on the cell type [62].

**Table 4:** Major compounds detected in *A. catechu* extract.

No.	Putative Name	% Abd.	Antibacterial Activity	Refs.
1	Arecoline	33.5	Probable	[63]
2	Nicotinyl alcohol	10.3	Probable	[64]
3	Guvacoline	5.8	n/a	-
4	Caffeine	3.2	Yes	10
5	Betaine	2.9	Probable	[65,66]
6	9-Oxo-10(E),12(E)-octadecadienoic acid	2.8	n/a	-
7	2-[(5Z)-5-tetradecenyl]cyclobutanone	2.7	n/a	-
8	Methyl isonicotinate	2.3	n/a	-
9	9,10-Dihydroxystearic acid	2.0	n/a	-
10	(±)12(13)-DiHOME	1.8	n/a	-
11	Stearamide	1.8	n/a	-
12	Guvacine	1.7	n/a	-
13	Cianidanol	1.5	Yes	[56]
14	Tranexamic acid	1.5	Yes	[57]
15	Phytosphingosine	1.3	Yes	[58]
16	Indane	1.2	n/a	-
17	Butyryl-L-homoserine lactone	1.2	n/a	-
18	Ethyl palmitoleate	1.1	n/a	-
19	L-α-Palmitin	1.0	n/a	-
20	Hexadecanamide	0.8	n/a	-
21	Protocatechuic acid	0.7	Yes	[61,62,67]
22	Nootkatone	0.7	Yes	[59,60]
23	α-Eleostearic acid	0.7	n/a	-

Note: n/a = not available.

## 3. Materials and Methods

### 3.1. Materials and Chemicals

*A. catechu* fruit powder was obtained from CV. Jaya Mulya Kimia, Bantul Regency, Yogyakarta, Indonesia. Coconut water waste was obtained from the Minggir subdistrict, Yogyakarta, Indonesia. Bacterial cellulose was synthesized using coconut water according to a prior study [68]. It was dried in an oven at 40 °C for 12 h and stored at 4 °C in the refrigerator. Carboxymethyl cellulose (CMC) was obtained from a local market, Yogyakarta Province, Indonesia. Amoxycillin antimicrobial susceptibility disks (Oxoid 10 µg) and pathogenic bacteria, i.e., *E. coli* (strain ATCC 8739) and *S. aureus* (strain ATCC 25923), were acquired from Thermo Scientific (Landsmeer, The Netherlands).

### 3.2. A. catechu Extraction

Dried powder of *A. catechu* fruit was extracted by maceration using ethanol at a ratio of 1:10 *w*/*v* at room temperature for 24 h. The mixture obtained was filtered through filter paper, and subsequently, the solvent was evaporated to yield the ethanol extract of *A. catechu*. The extracts were then stored in the refrigerator at a temperature of 4 °C.

### 3.3. Film Composite Preparation

Ag-BC-*A. catechu* composite films were synthesized according to previous studies with several modifications [69]. BC film sheets (thickness 0.015 mm) were impregnated with silver nitrate solution (100 mL, 0.01 M) for 60 min at room temperature. Ag^+^-BC was then reacted with sodium citrate (10 mL, 1 M) at 100 °C for 1 h to reduce silver ions, Ag^+^ into Ag^0^. In the next step, the Ag/BC composite film formed was then impregnated with *A. catechu* fruit extract (10^−3^-mg/mL) for 5 h at room temperature. Table 5 displays various concentrations of *A. catechu* fruit extract. The composite films produced were allowed to dry at room temperature for 24 h and were subsequently stored in the refrigerator until they were ready for further analysis (Figure S4).

### 3.4. Morphology Characterization

The morphology of the film was examined utilizing an SEM (SU-3500, Hitachi, Tokyo, Japan) [32]. Images of all samples were obtained using an accelerating voltage of 3.00 kV and a magnification of 10.0k.

### 3.5. XRD Characterization

The film’s structure and crystallinity were analyzed through XRD (Rigaku Smartlab, Tokyo, Japan). Prior to the analysis, the films were sectioned into 2 cm × 2 cm dimensions. The device was used at 30 mA and 40 kV, using Cu-Kα radiation (λ = 1.5418 Å). The crystallite size and Segal crystallinity index (CI) were calculated from the diffraction pattern obtained from experimental data. The crystallite size, D (nm), perpendicular to the lattice plane (200) for cellulose I was calculated using the Scherrer equation (Equation (1)), as described by Nam et al. [70].

(1) $$D=\frac{K\lambda }{\beta \cos\theta }$$

where K is the correction factor (0.9), λ is the wavelength of X-ray radiation (1.5406 Å), β is the FWHM of the diffraction peak (radians), and θ is the diffraction angle of the peak. The Segal CI (%) was calculated using Equation (2).

(2) $$\mathrm{CI}=\frac{I_{t}-I_{a}}{I_{t}}\times 100$$

where I_t_ is the total peak intensity of the lattice plane (200) at 22.7°, and I_a_ is the amorphous intensity at 18° for cellulose I.

### 3.6. FTIR Spectroscopy

The FTIR spectroscopy method was used to evaluate the functional groups of the films (Vertex 80, Bruker, Ettlingen, Germany) [32]. FTIR acquisition was performed for each sample within the wavelength range of 4000–500 cm^−1^, utilizing a resolution of 4 cm and conducting 32 scans.

### 3.7. Swelling Capacity Analysis

The film’s swelling capacity was analyzed using the previous method [32]. The composites were measured for weight both before and after being immersed in 30 mL of water for 24 h at room temperature. This investigation utilized replication with three parallel samples. The swelling capacity percentage was calculated using the equation below:

(3) $$\%\;\mathrm{swelling}\;\mathrm{capacity}\;=\;\left(\frac{W_{s}\;-\;W_{i}}{W_{i}}\right)\times 100$$

W_i_ and W_s_ correspond to the film weights before and after 24 h of water soaking, respectively.

### 3.8. Color Measurement

A Konica Minolta Color Reader (Tokyo, Japan) was used to assess the film’s color (CR-20). CIE color values (L*, a*, b*) were documented, where L* represents lightness to darkness, a* indicates the spectrum from green to red, and b* ranges from blue to yellow.

### 3.9. Antibacterial Activity Using Disk-Diffusion Method

The antibacterial properties of the film were assessed against two bacterial strains: *E. coli* and *S. aureus* [71]. Each film was formed into a square with a side of 10 mm. The films were arranged on a Mueller-Hinton Agar (MHA) medium plate that contained 10^8^ CFU/mL of bacteria, which aligns with a 0.5 McFarland standard. An amoxicillin disk (10 μg, 5 mm in diameter) was also placed on the agar plates. Subsequently, the plates were incubated for 24 h at a temperature of 37 °C. Following the incubation period, the diameters of the inhibition zones were measured to evaluate the antibacterial efficacy of both the samples and the controls. The reported inhibition-zone values represent the total diameter of the clear inhibition zone minus the diameter of the test disk or sample, so it does not include the 5 mm diameter test disk or 10 mm of sample, as measured from one outer edge of the inhibition zone to the opposite outer edge.

The final of inhibiton All measurements were documented as the average of three independent replicates.

### 3.10. LC-HRMS Analysis

The LC-HRMS analysis was carried out based on prior investigations [72]. The Thermo Scientific™ Vanquish™ UHPLC Binary Pump and the Orbitrap high-resolution mass spectrometer (Germering, Germany) were utilized to conduct liquid chromatography-high-resolution mass spectrometry (LC-HRMS). An extract weighing 5 mg was dissolved in 1 mL of methanol and vortexed for 30 s. The sample was then filtered into an LC-HRMS vial using a 0.22 μm nylon filter. The analysis was carried out with an Accucore phenyl-hexyl column (100 mm × 2.1 mm × 2.6 μm) by injecting 5 μL of the samples, which were eluted with water (A) and acetonitrile (B), both containing 0.1% formic acid, according to the following gradient: 0–5 min (5% B), 5–16 min (5–90% B), 16–20 min (90% B), and 20–25 min (90–5% B). The temperature was adjusted to 8 °C for the sampler and 40 °C for the column. Compounds were identified using an Orbitrap HRMS, with a sheath gas flow rate of 32 AU and an auxiliary gas flow rate of 8 AU. The scanning of compounds was performed in both positive and negative ionization modes, covering a scan range of 66.7–1000 *m*/*z*, with resolutions set at 70,000 (MS1) and 14,500 (MS2). The capillary temperature was kept constant at 320 °C. Additionally, the unprocessed chromatograms underwent examination utilizing the Compound Discoverer 3.2 (Thermo Scientific™) software, referencing both local and online databases, which included mzCloud (www.mzcloud.org) and ChemSpider (www.chemspider.com). The criteria for analyzing the metabolite profiles were defined as follows: potential compounds were selected according to their alignment with the fragmentation patterns found in mzCloud, and the deviation in their molecular weight did not exceed 2 ppm.

### 3.11. Statistical Analysis

In this study, each experiment was carried out three times and then put through statistical analysis using one-way ANOVA, followed by Duncan’s multiple range test in IBM SPSS Statistics 27. The differences between the samples were deemed statistically significant at *p* < 0.05.

## 4. Conclusions

This study successfully developed a sustainable Ag-BC-*A catechu* extract nanocomposite biofilm with potential for further development in active food packaging applications. The incorporation of silver nanoparticles and *A. catechu* extract into the BC matrix resulted in a multifunctional biofilm with enhanced physicochemical and antibacterial properties. Comprehensive characterization confirmed the successful formation of the nanocomposite and demonstrated favorable structural, morphological, and functional characteristics. Furthermore, the biofilm exhibited significant antibacterial activity against *S. aureus* and *E. coli*, highlighting the synergistic antimicrobial effects of silver nanoparticles and the bioactive phytochemicals present in *A. catechu* extract. These findings suggest that the developed nanocomposite biofilm represents an environmentally friendly alternative to conventional petroleum-based food packaging materials. However, this study did not evaluate the mechanical strength of the films or the migration of silver/*A. catechu* constituents into food or food simulants. Therefore, the food-contact safety and suitability of the films have not yet been established. Future work should therefore incorporate mechanical strength, biodegradation behavior, and effectiveness in real food storage systems to facilitate its promising application in the food packaging industry. Additional toxicological and food-contact safety assessments will also be required to confirm the composite film’s suitability for food packaging.

## Figures and Tables

**Figure 1:**
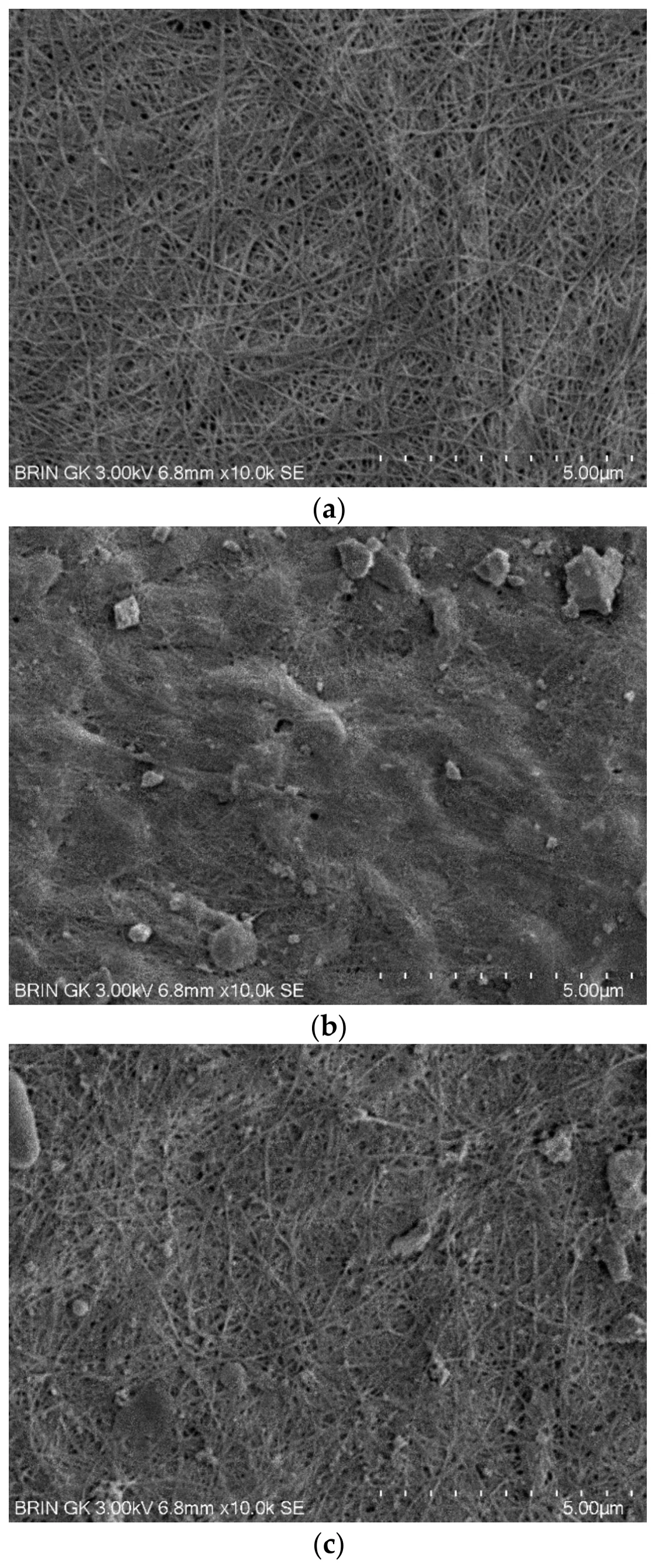

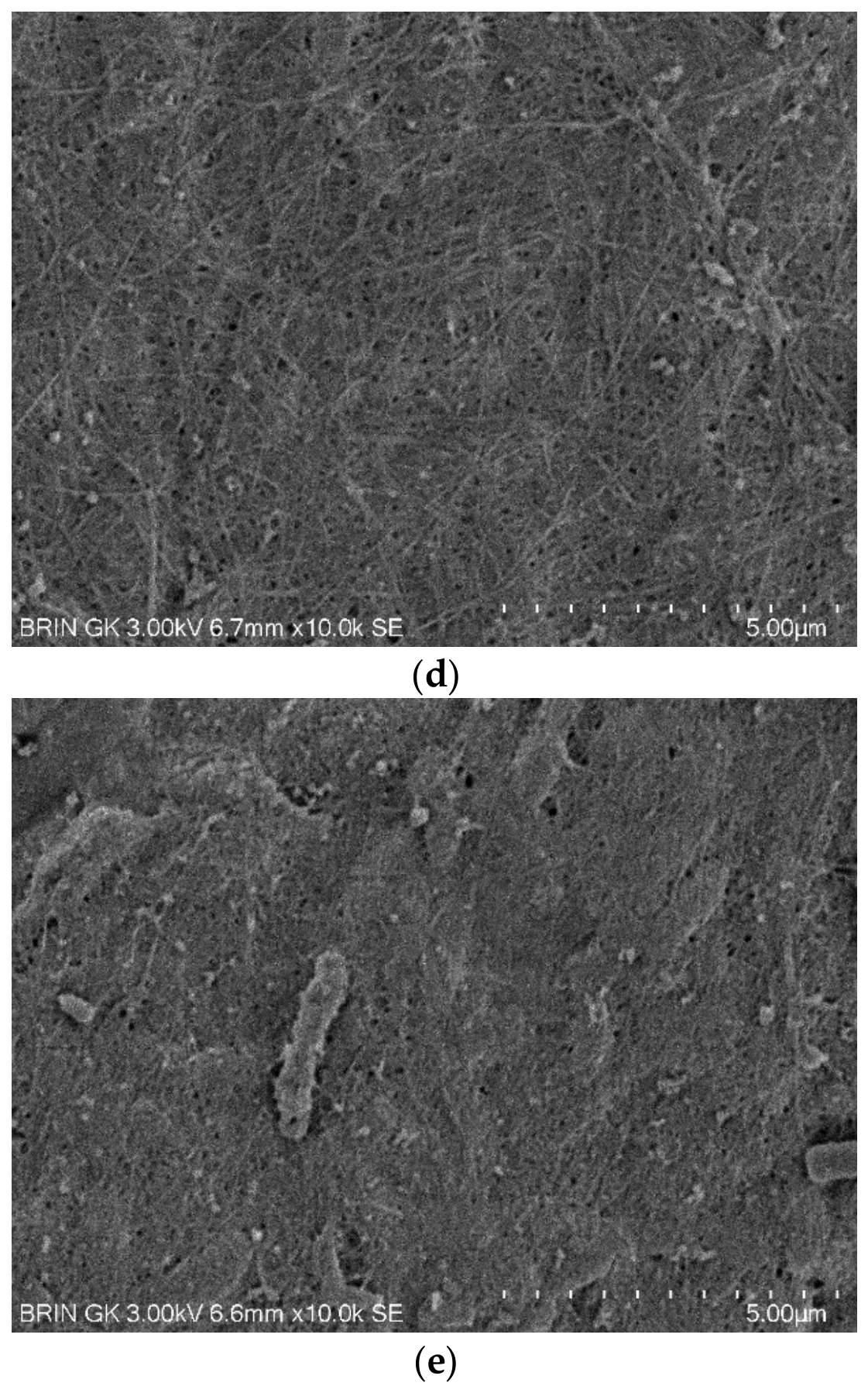
SEM images of (**a**) pure bacterial cellulose (BC); (**b**) Ag-BC (without *A. catechu* fruit extract); (**c**) Ag-BC-*A. catechu* 1 (film with 10 mg/mL *A. catechu* fruit extract); (**d**) Ag-BC-*A. catechu* 2 (film with 20 mg/mL *A. catechu* fruit extract); (**e**) Ag-BC-*A. catechu* 3 (film with 30 mg/mL *A. catechu* fruit extract).

**Figure 2:**
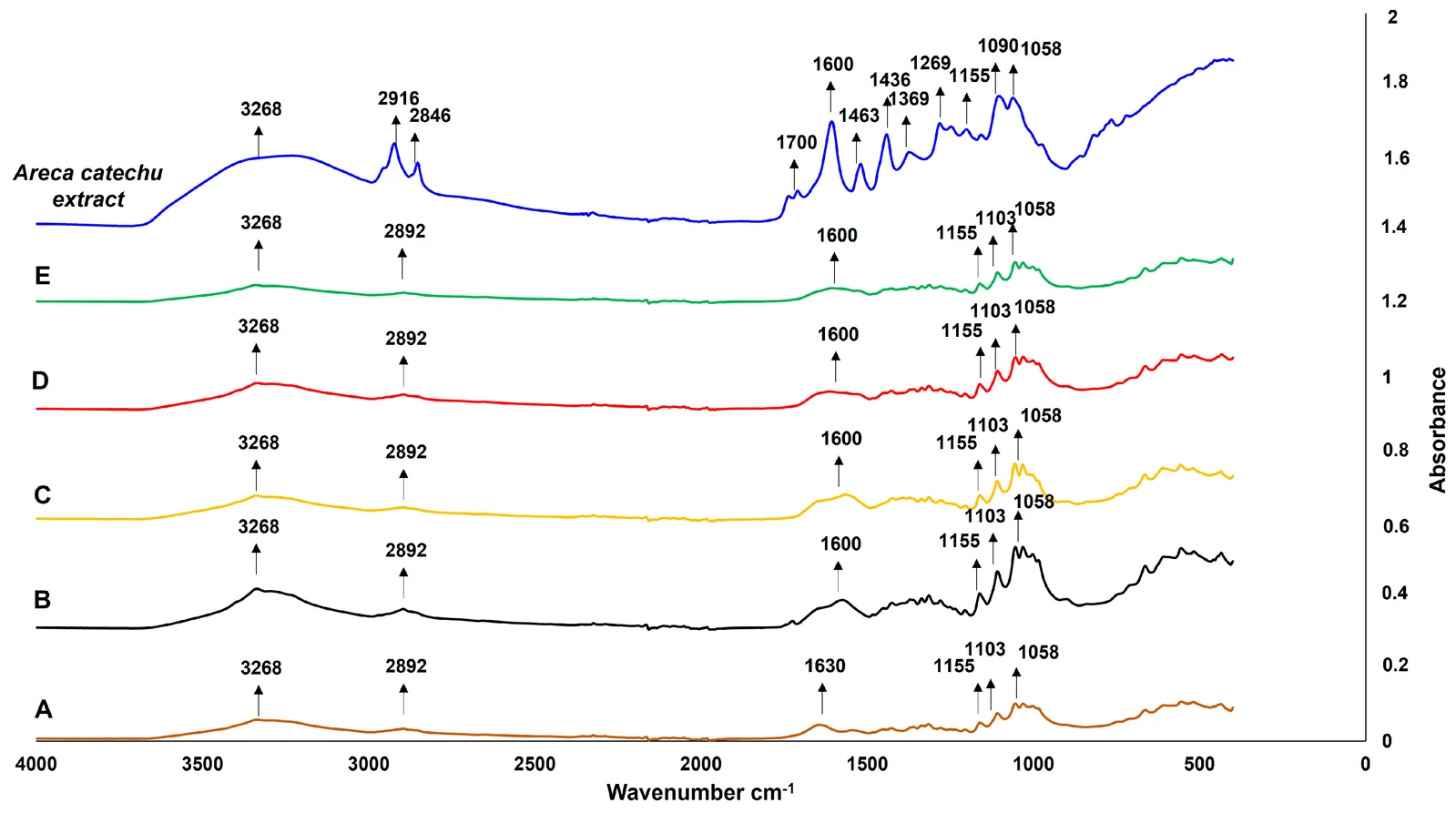
FTIR spectra of *A. catechu extract*, pure bacterial cellulose (A); Ag-BC without *A. catechu* fruit extract (B); Ag-BC-*A. catechu* 1 (film with 10 mg/mL *A. catechu* fruit extract) (C); Ag-BC-*A. catechu* 2 (film with 20 mg/mL *A. catechu* fruit extract) (D); Ag-BC-*A. catechu* 3 (film with 30 mg/mL *A. catechu* fruit extract) (E).

**Figure 3:**
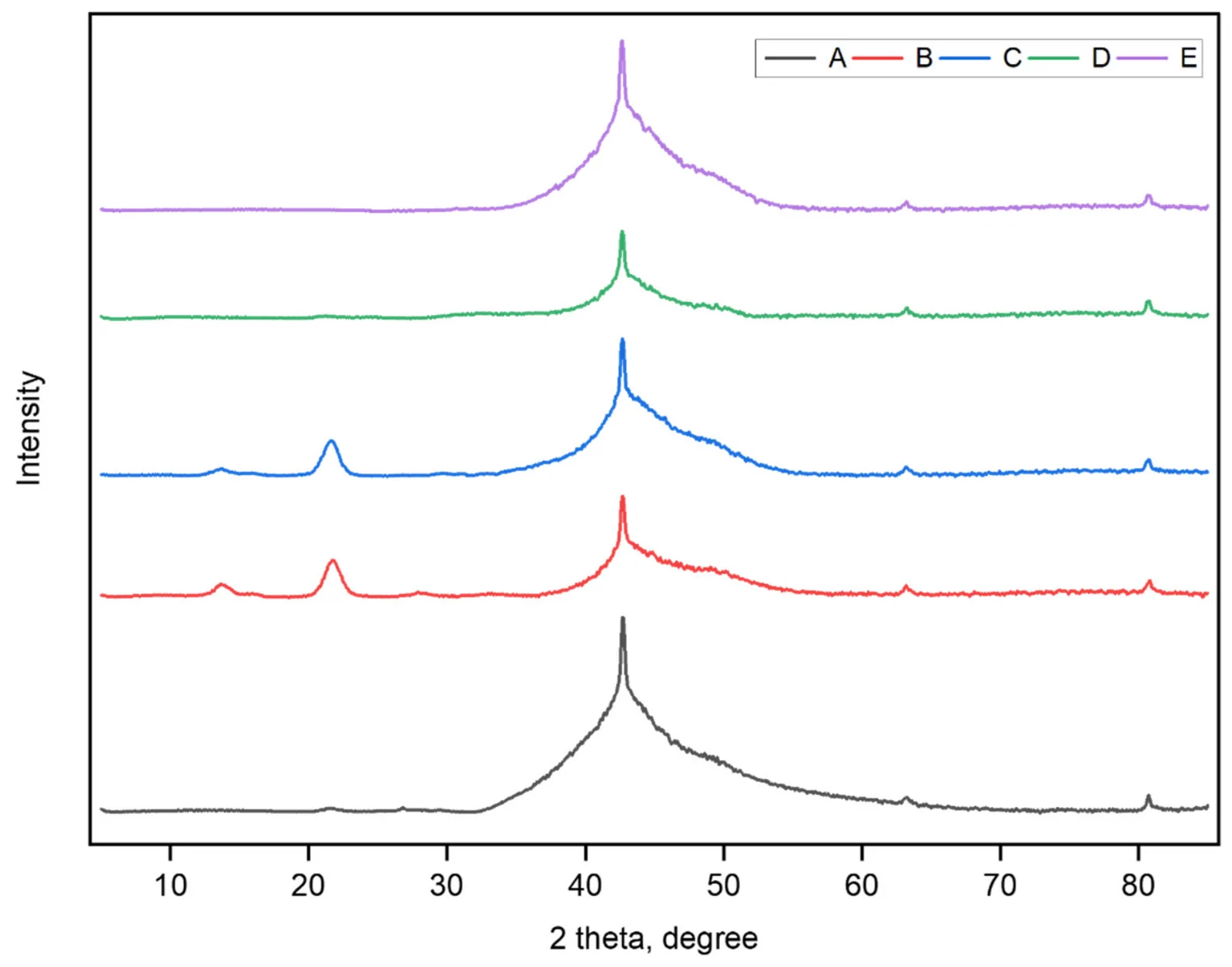
XRD diffractogram of *A. catechu extract*, pure bacterial cellulose (A); Ag-BC without *A. catechu* fruit extract (B); Ag-BC-*A. catechu* 1 (film with 10 mg/mL *A. catechu* fruit extract) (C); Ag-BC-*A. catechu* 2 (film with 20 mg/mL *A. catechu* fruit extract) (D); Ag-BC-*A. catechu* 3 (film with 30 mg/mL *A. catechu* fruit extract) (E).

**Figure 4:**
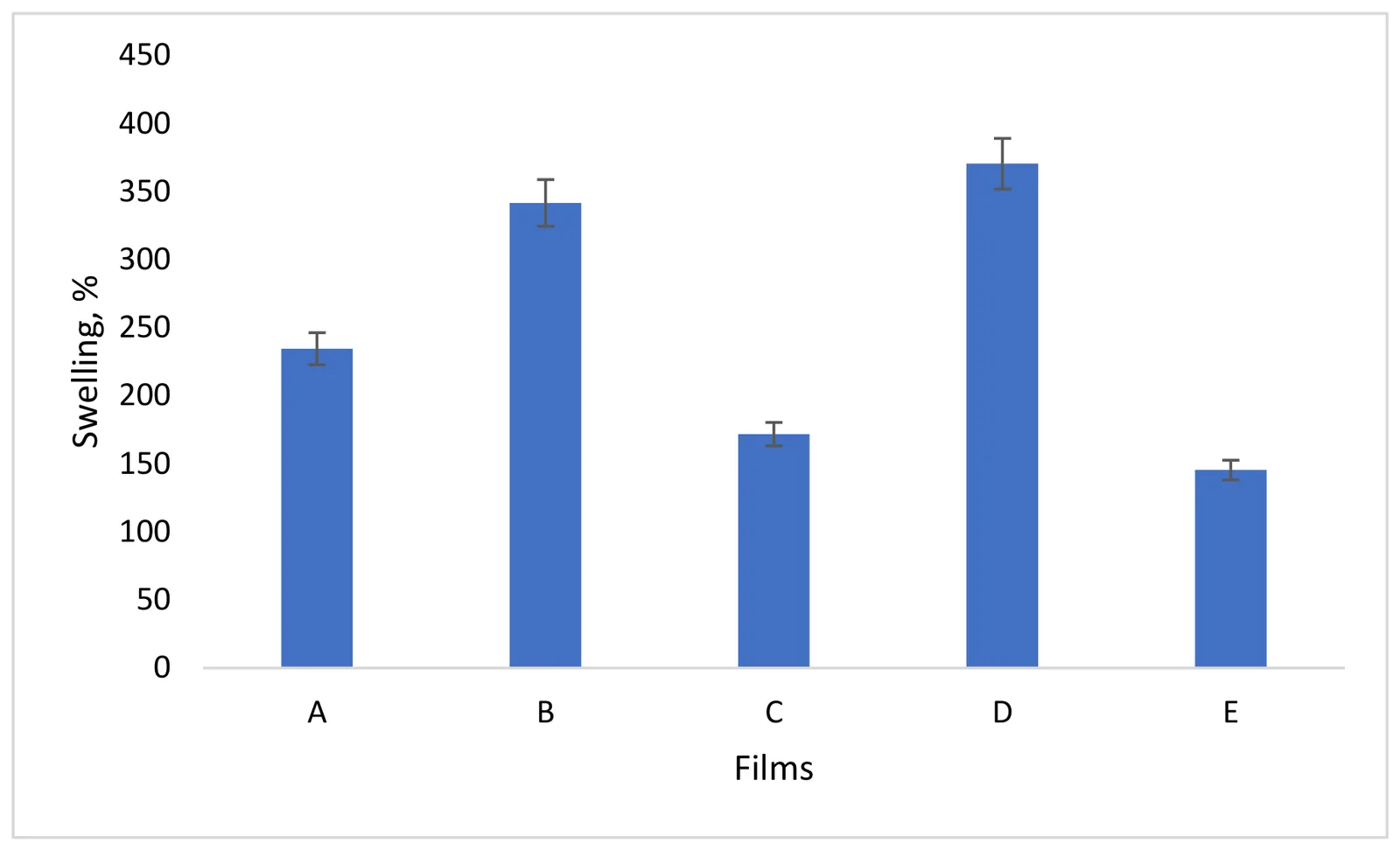
Swelling capacity of Ag-BC films: A. pure bacterial cellulose (BC); B. Ag-BC (without *A. catechu* fruit extract); C. Ag-BC-*A. catechu* 1 (film with 10 mg/mL *A. catechu* fruit extract); D. Ag-BC-*A. catechu* 2 (film with 20 mg/mL *A. catechu* fruit extract); E. Ag-BC-*A. catechu* 3 (film with 30 mg/mL *A. catechu* fruit extract).

**Table 1:** Elemental composition of Ag-BC-AC films observed using EDX spectroscopy.

Element (%)	A	B	C	D	E
C	54.1	57.7	61.8	54.3	51.7
O	45.1	35.5	30.6	43.3	44.2
Na	0.3	5.9	7.1	1.8	3.3
P	0.3	0.1	0.2	0.2	0.2
S	0.1	0.1	0.1	0.1	0.1
Ag	0	0.6	0.1	0.3	0.3
K	0.1	0	0	0	0.1
Ca	0	0.1	0.1	0	0.1
Total	100	100	100	100	100

Notes: Pure bacterial cellulose (A); Ag-BC without *A. catechu* fruit extract (B); Ag-BC-*A. catechu* 1 film with 10 mg/mL *A. catechu* fruit extract (C); Ag-BC-*A. catechu* 2 film with 20 mg/mL *A. catechu* fruit extract (D); Ag-BC-*A. catechu* 3 film with 30 mg/mL *A. catechu* fruit extract (E).

**Table 2:** Color analysis of Ag-BC-*A. catechu* films.

Films	L*	a*	b*
A	43.25 ± 0.34 ^d^	−0.54 ± 0.03 ^a^	−0.04 ± 0.18 ^a^
B	30.80 ± 0.10 ^a^	2.95 ± 0.24 ^b^	8.93 ± 0.12 ^b^
C	35.16 ± 0.22 ^c^	6.60 ± 0.16 ^c^	12.72 ± 0.55 ^e^
D	31.50 ± 0.33 ^b^	6.72 ± 0.15 ^c^	12.03 ± 0.20 ^d^
E	35.42 ± 0.18 ^c^	6.42 ± 0.90 ^c^	10.48 ± 0.38 ^c^

Note: pure bacterial cellulose (A); Ag-BC without *A. catechu* fruit extract (B); Ag-BC-*A. catechu* 1 (film with 10 mg/mL *A. catechu* fruit extract) (C); Ag-BC-*A. catechu* 2 (film with 20 mg/mL *A. catechu* fruit extract) (D); Ag-BC-*A. catechu* 3 (film with 30 mg/mL *A. catechu* fruit extract) (E). Values with different superscript letters within the same column are significantly different (*p* < 0.05), whereas values sharing the same superscript letter are not significantly different.

**Table 3:** Antibacterial assay of Ag-BC-*A. catechu* films.

Films	*S. aureus* (mm)	*E. coli* (mm)
A	0 ^a^	0 ^a^
B	4.62 ± 0.12 ^d^	3.83 ± 0.4 ^b^
C	6.50 ± 0.6 ^e^	6.50 ± 0.5 ^d^
D	2.50 ± 0.2 ^b^	3.33 ± 0.1 ^b^
E	3.25 ± 0.4 ^c^	4.25 ± 0.3 ^c^
Amoxycillin	16.75 ± 0.1 ^f^	16.50 ± 0.2 ^e^

Note: pure bacterial cellulose (A); Ag-BC without *A. catechu* fruit extract (B); Ag-BC-*A. catechu* 1 (film with 10 mg/mL *A. catechu* fruit extract) (C); Ag-BC-*A. catechu* 2 (film with 20 mg/mL *A. catechu* fruit extract) (D); Ag-BC-*A. catechu* 3 (film with 30 mg/mL *A. catechu* fruit extract) (E). Values with different superscript letters within the same column differ significantly (*p* < 0.05), whereas values sharing the same superscript letter are not significantly different.

**Table 5:** Ag-BC-*A. catechu* composite biofilm formula.

Films	Ag^+^ Concentration (M)	*A. catechu* Fruit Extract Concentration (mg/mL)
A (pure BC)	-	-
B (Ag-BC)	0.01	-
C (Ag-BC-*A. catechu* 1)	0.01	10
D (Ag-BC-*A. catechu* 2)	0.01	20
E (Ag-BC-*A. catechu* 3)	0.01	30

## Data Availability

Data is contained within the article or Supplementary Materials.

## Supplementary Materials

The following supporting information can be downloaded at: https://www.mdpi.com/article/10.3390/molecules31193373/s1, Figure S1: Energy-dispersive X-ray spectrometric (EDX) spectra of films; Figure S2: The EDS map of the silver distribution of film Ag-BC-*A. catechu*; Figure S3: The representative photographs of the antibacterial plates of Ag-BC-*A. catechu* film against *E. coli* (a) and *S. aureus* (b); Figure S4: Physical appearance of films; Table S1: Physical parameters estimated from XRD analysis; Table S2: Metabolite compounds of *A. catechu* extract; Table S3: Fragmentation profiles of selected compounds.

## Abbreviations

The following abbreviations are used in this manuscript:

BC	Bacterial cellulose
SEM-EDX	Scanning electron microscopy-energy-dispersive X-ray spectroscopy
FTIR	Fourier-transform infrared
XRD	X-ray diffraction
AgNPs	Silver nanoparticles
LC-HRMS	Liquid chromatography-high resolution mass spectrometry
CMC	Carboxymethyl cellulose
MHA	Mueller Hinton Agar
CI	Crystallinity index
ROS	Reactive oxygen species
