# Supplementary material for: Silver-Bacterial Cellulose-Areca catechu Extract Nanocomposite Biofilm for Sustainable Food Packaging: Synthesis, Characterization, and Antibacterial Activity
Source: Molecules. 2026 Sep 23;31(19):3373. doi: 10.3390/molecules31193373 (PMC13665780; doi:10.3390/molecules31193373)
Supplement: Supplementary file 1 [file molecules-31-03373-s001.zip › molecules-4562319-supplementary.pdf]

Supplementary Information

a.

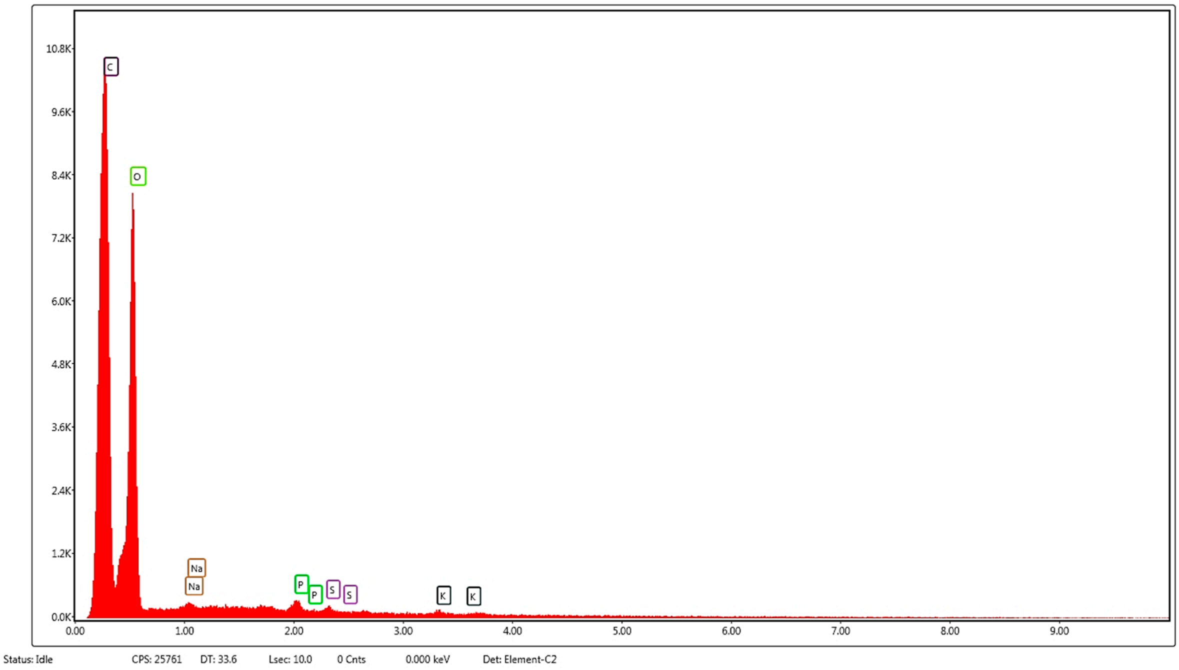

b.

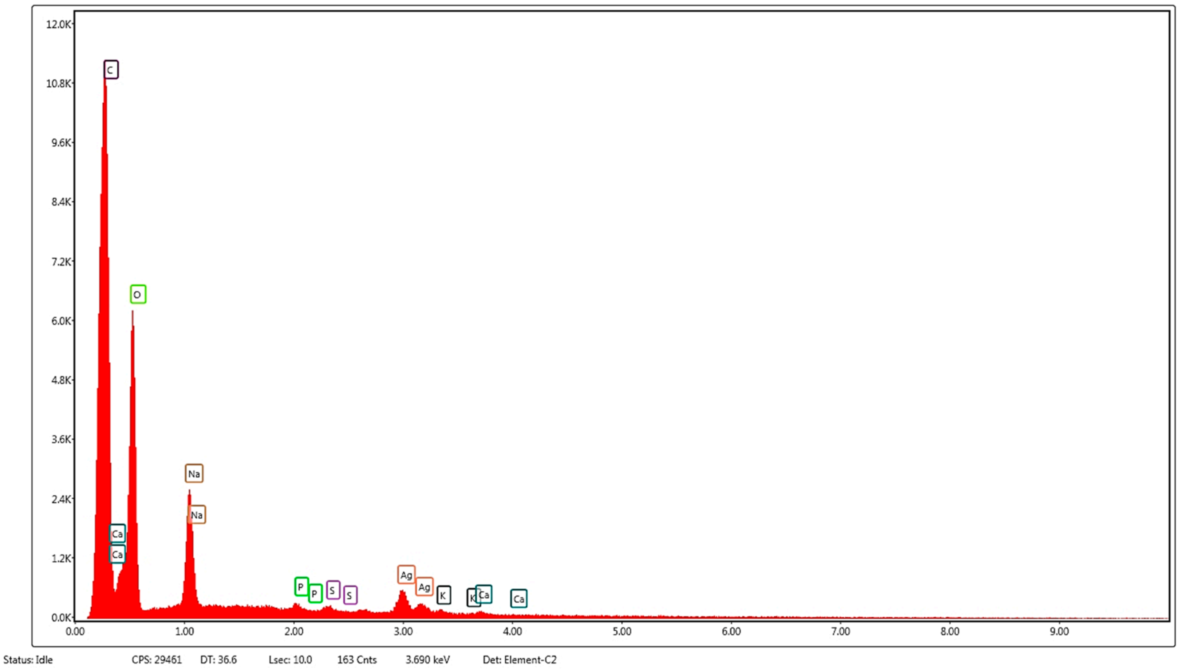

c.

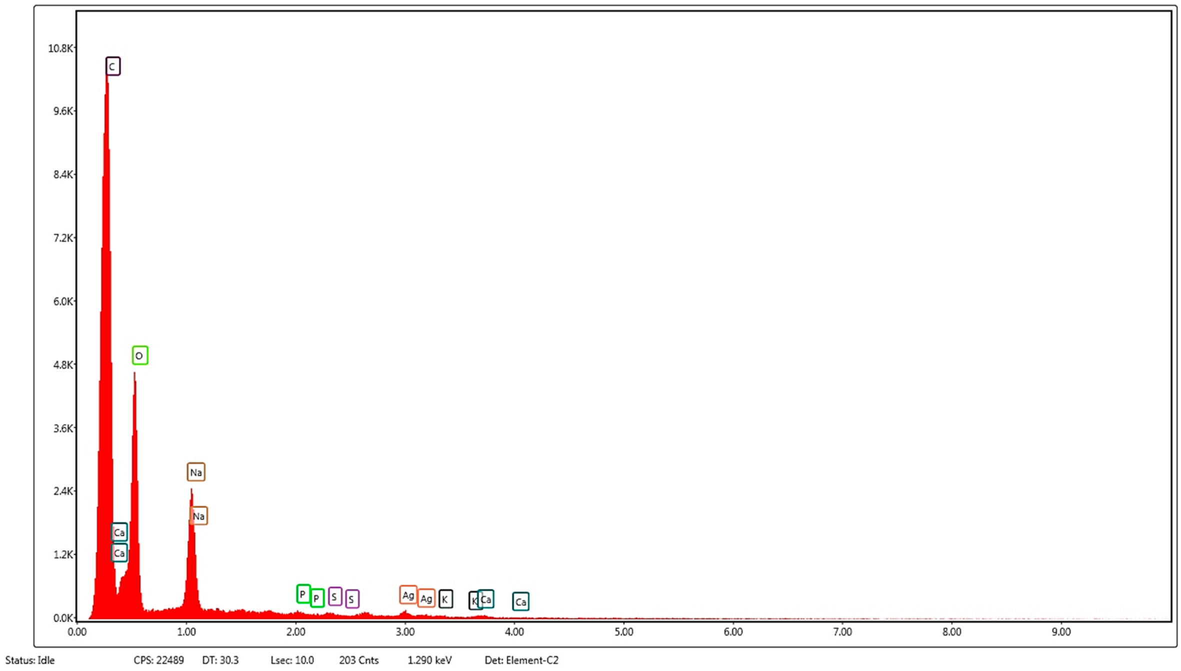
C
O
Na
Na
Ca
Ca
P P S S Ag Ag K K Ca Ca
10.8K
9.6K
8.4K
7.2K
6.0K
4.8K
3.6K
2.4K
1.2K
0.0K
0.00 1.00 2.00 3.00 4.00 5.00 6.00 7.00 8.00 9.00
Status: Idle CPS: 22489 DT: 30.3 Lsec: 10.0 203 Cnts 1.290 keV Det: Element-C2

d.

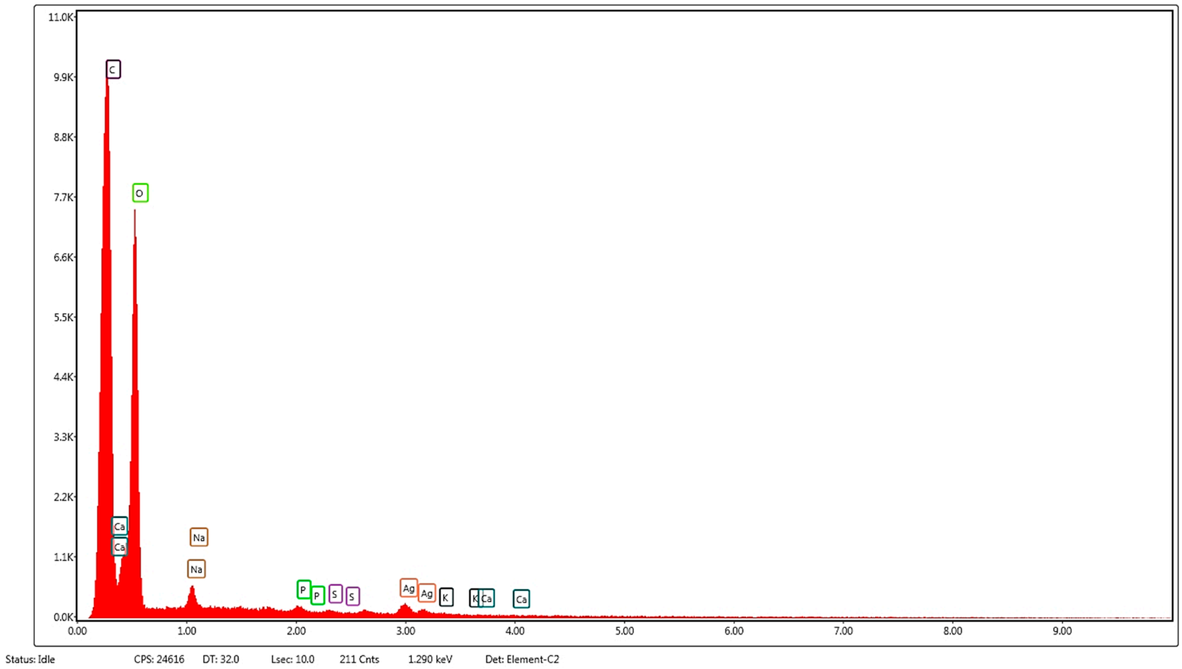
C
O
Ca
Ca
Na
Na
P P S S Ag Ag K K Ca Ca
11.0K
9.9K
8.8K
7.7K
6.6K
5.5K
4.4K
3.3K
2.2K
1.1K
0.0K
0.00 1.00 2.00 3.00 4.00 5.00 6.00 7.00 8.00 9.00
Status: Idle CPS: 24616 DT: 32.0 Lsec: 10.0 211 Cnts 1.290 keV Det: Element-C2

e

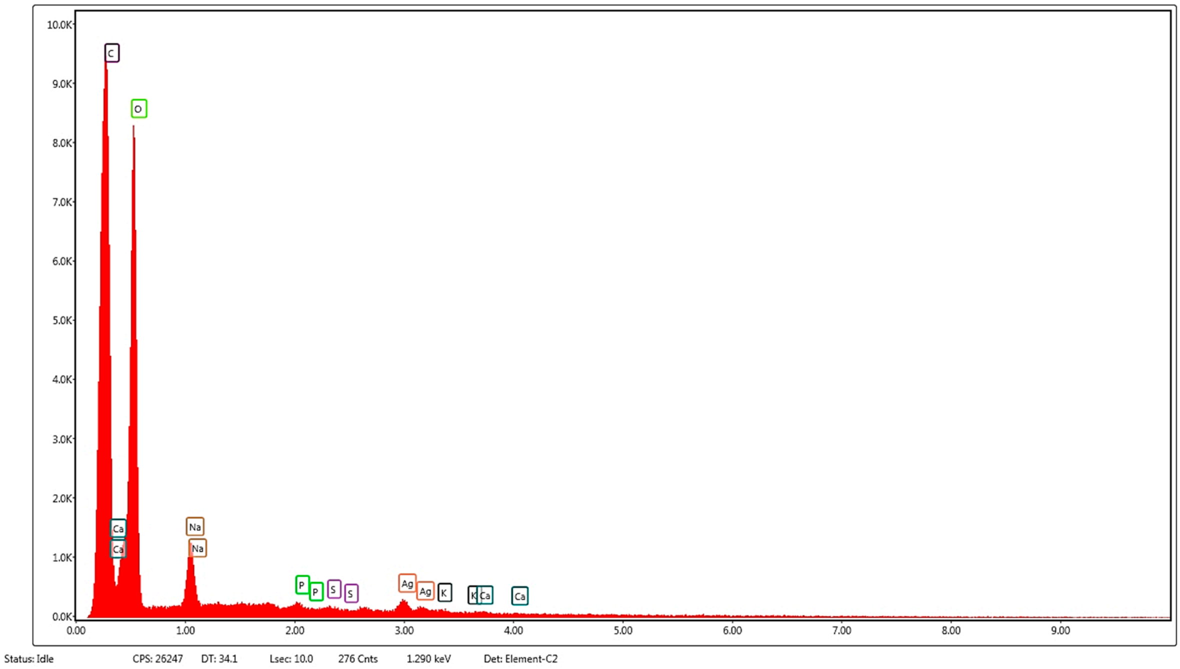

Figure S1. Energy dispersive X-ray spectrometric (EDX) spectra of film Ag-BC-*A. catechu*: a. pure bacterial cellulose (BC); b. Ag-BC (without *A. catechu* fruit extract); c. Ag-BC-AC1 (film with 10 mg/ml *A. catechu* fruit extract); d. Ag-BC-AC2 (film with 20 mg/ml *A. catechu* fruit extract); e. Ag-BC-AC3 (film with 30 mg/ml *A. catechu* fruit extract).

a. 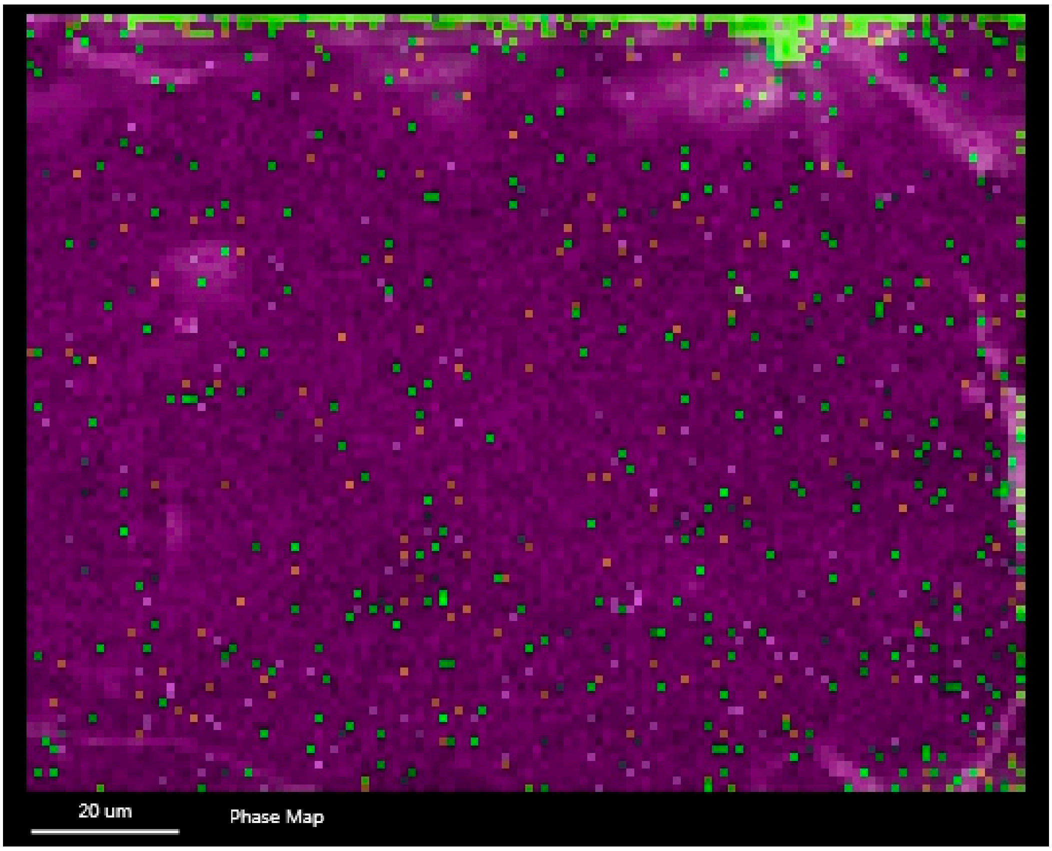

b. 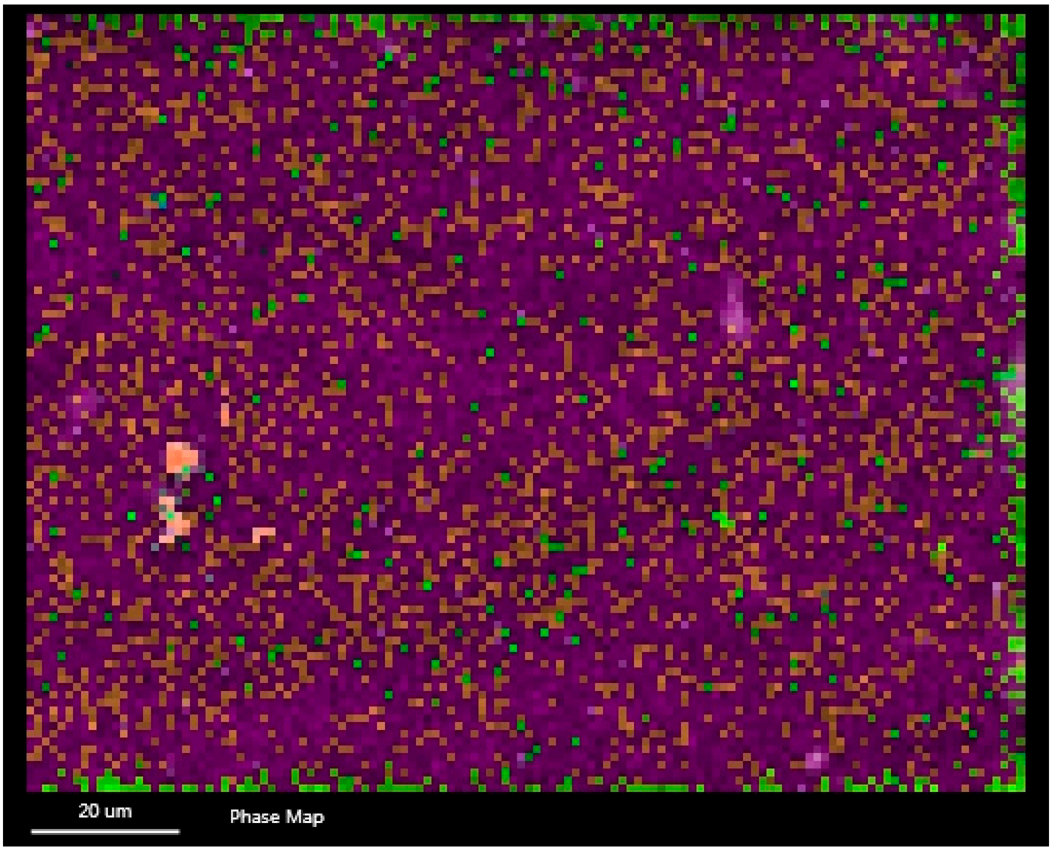

c.

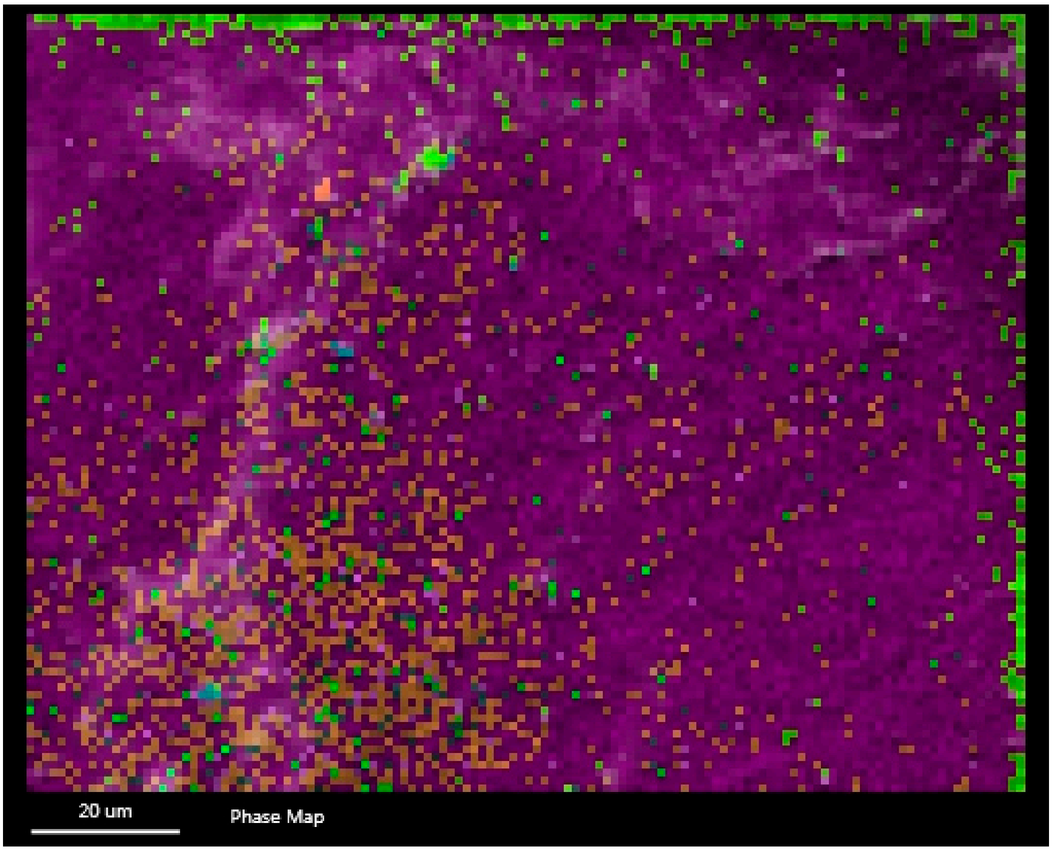

d.

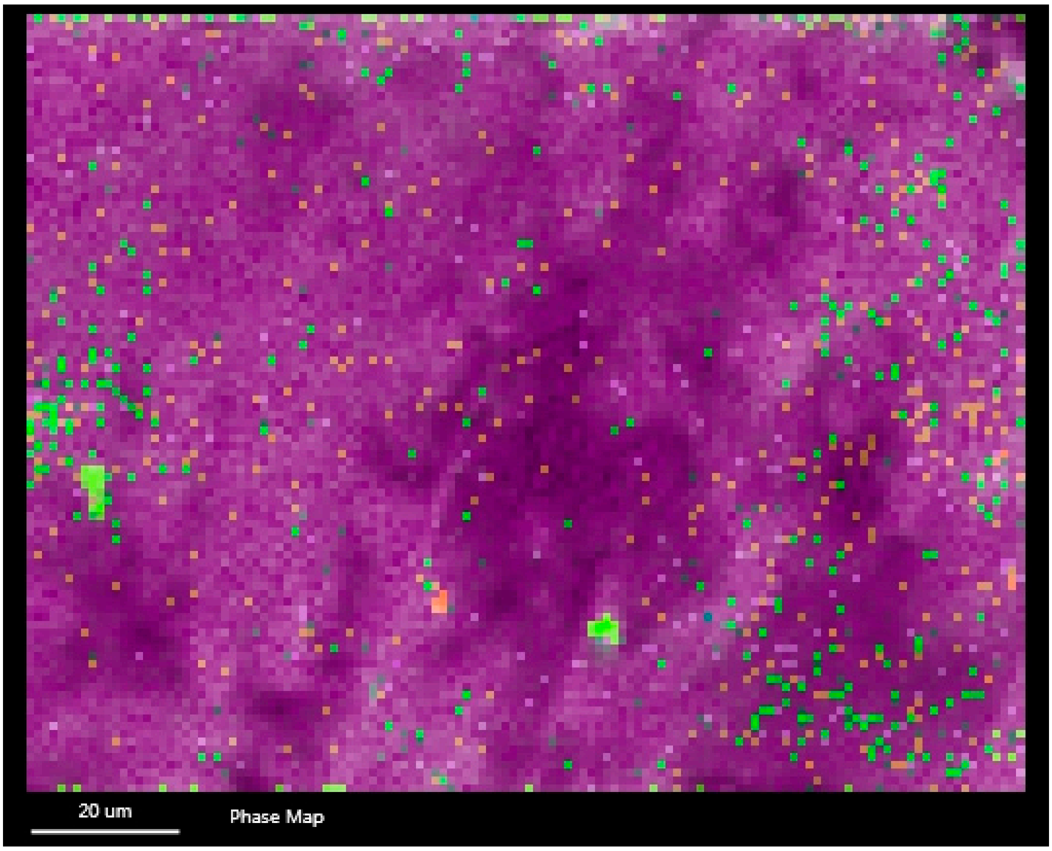

e. 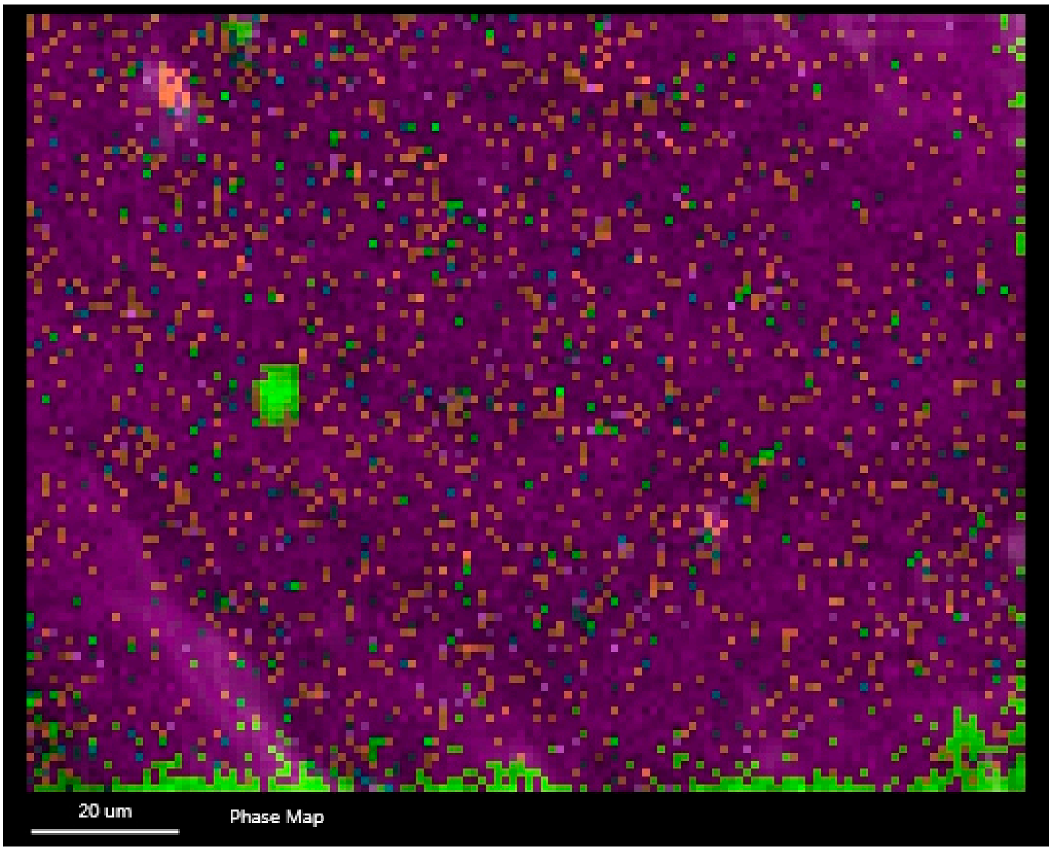

f. 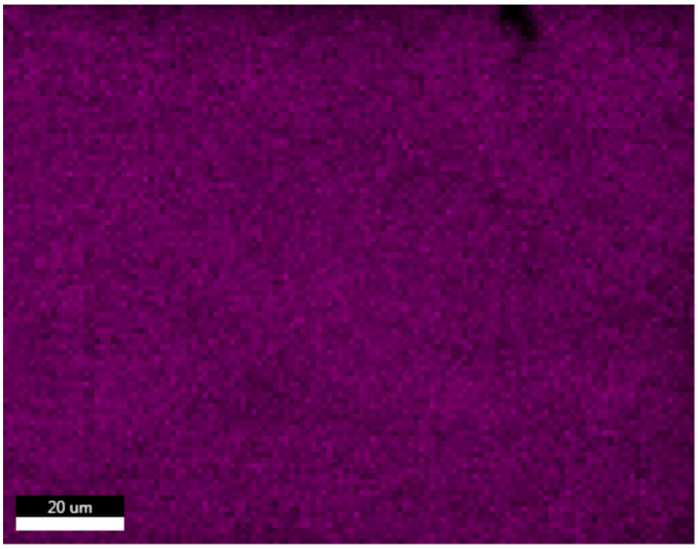

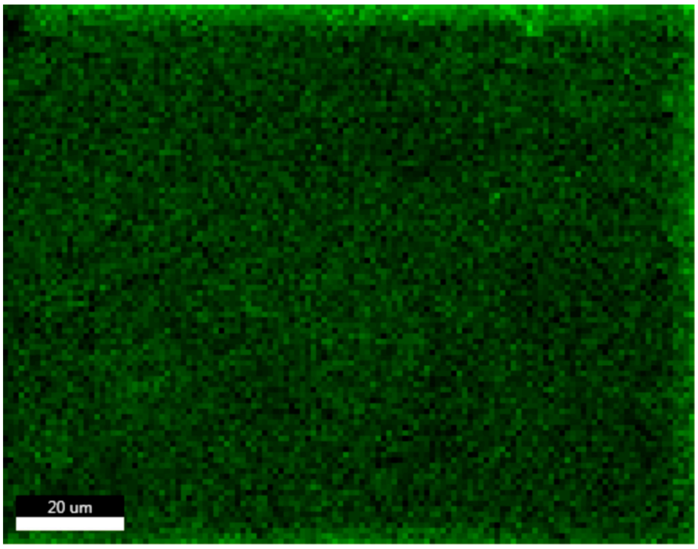

g.

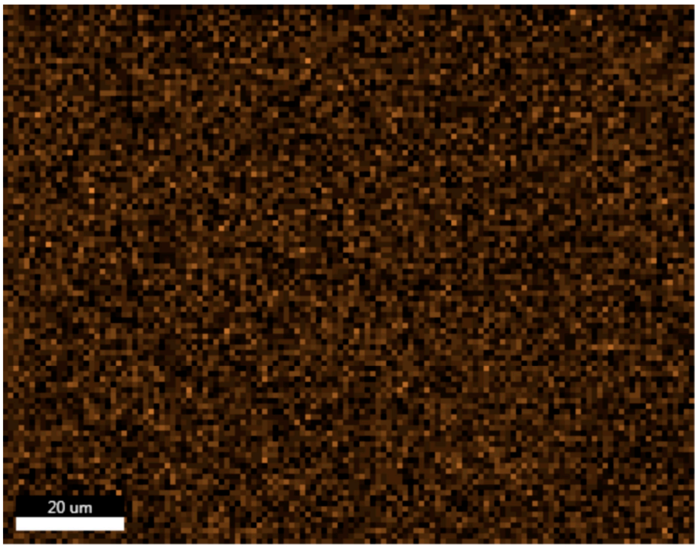

h.

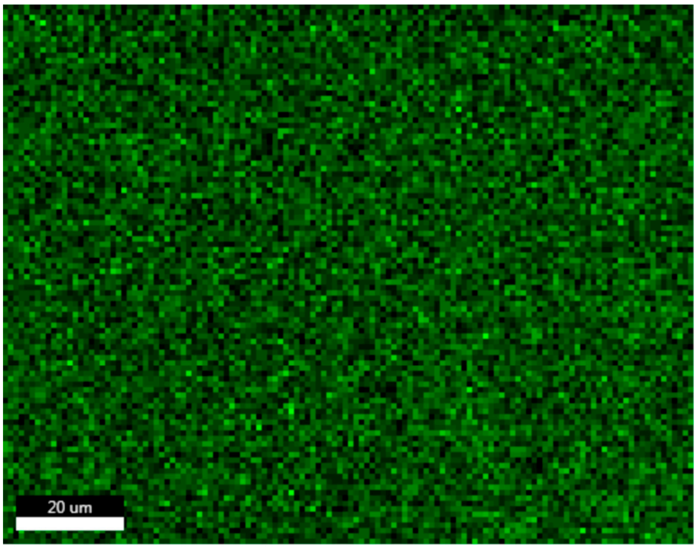

i.

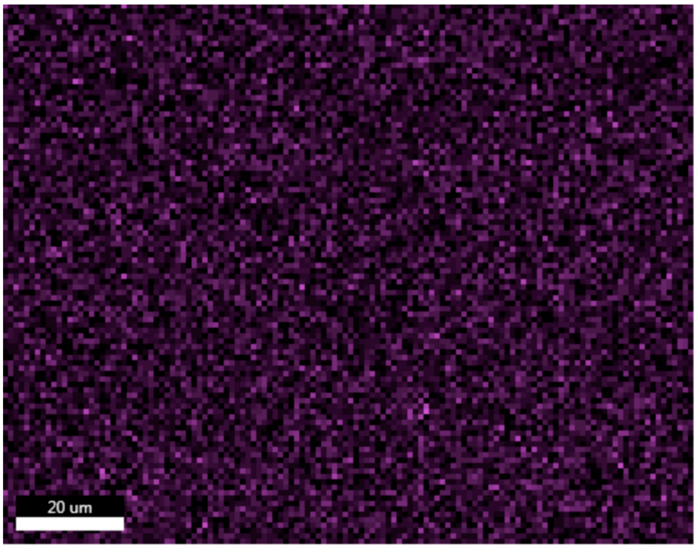

j.

k. 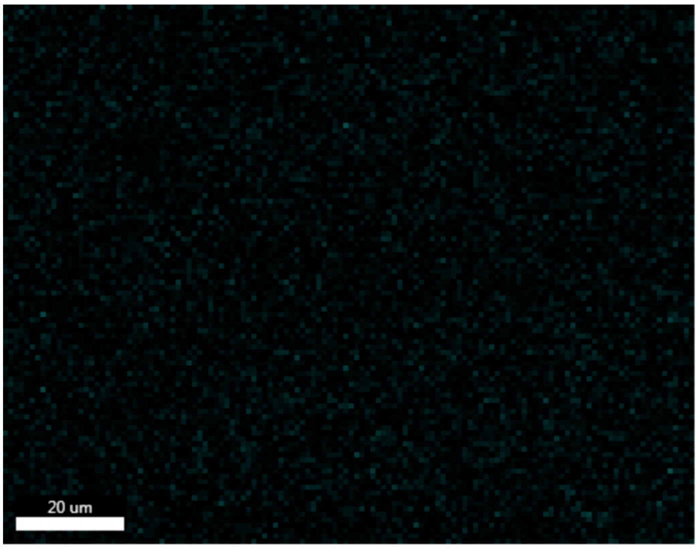

l. 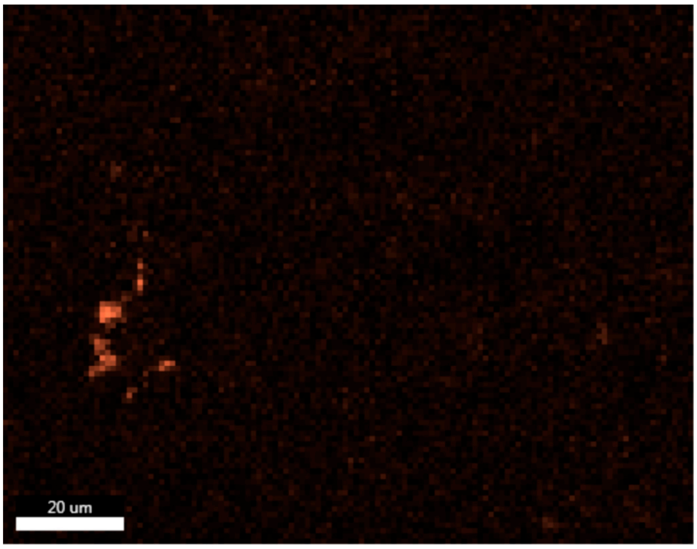

m. 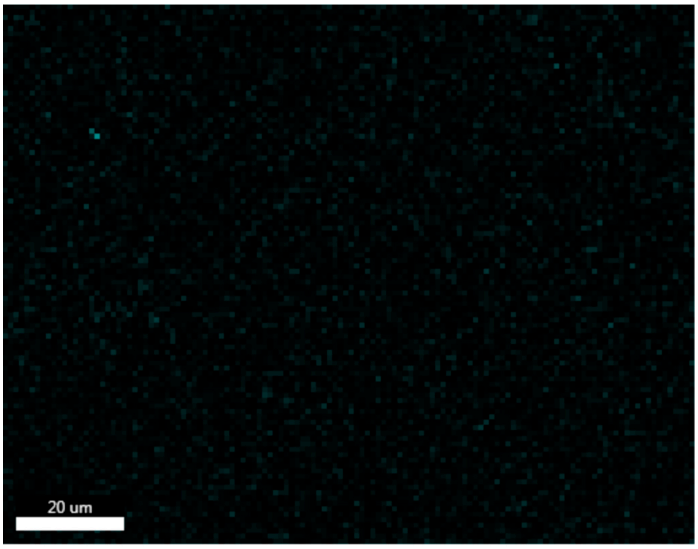

Figure S2. The EDS map of the silver distribution of film Ag-BC-*A. catechu*: a. pure bacterial cellulose (BC); b. Ag-BC (without *A. catechu* fruit extract); c. Ag-BC-AC1 (film with 10 mg/ml *A. catechu* fruit extract); d. Ag-BC-AC2 (film with 20 mg/ml *A. catechu* fruit extract); e. Ag-BC-AC3 (film with 30 mg/ml *A. catechu* fruit extract). The element color's mapping is provided as follows: f. C element, g. O element, h. Na element, i. P element, j. S element, k. K element, l. Ag element. m. Ca element.

a. (1) 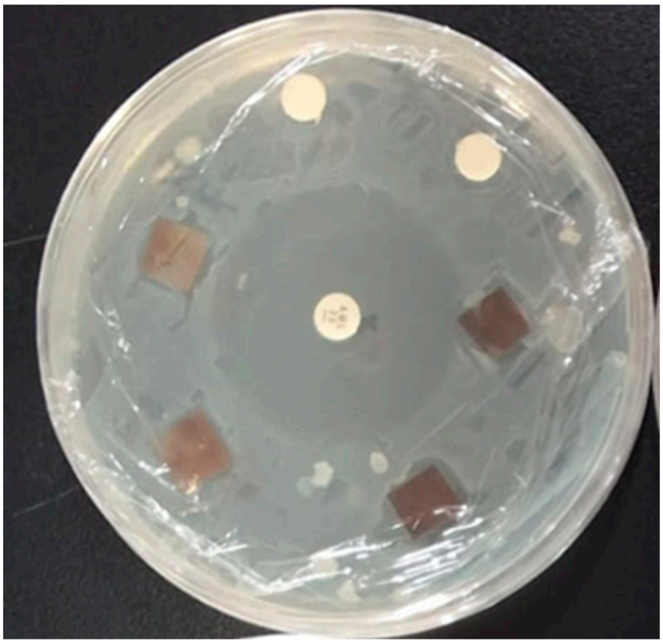 a. (2) 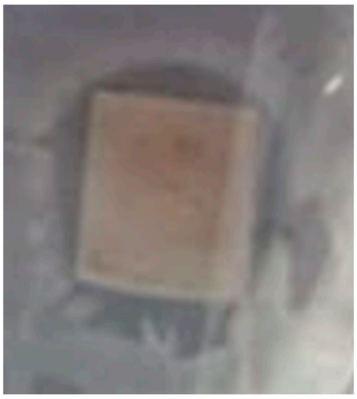

b. (1) 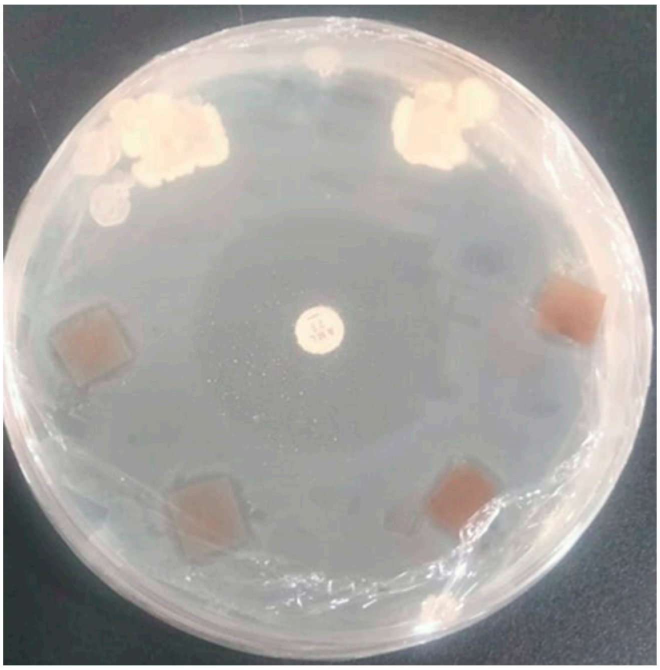 b. (2) 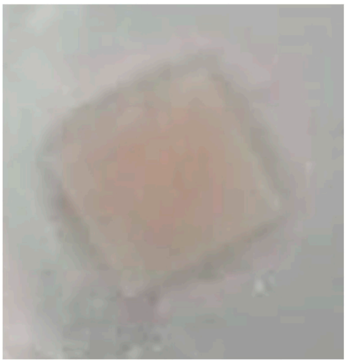

Figure S3. The representative photographs of the antibacterial plates of Ag-BC- *A. catechu* film against *E. coli* (a.1: full plate image a.2: one magnified representative image from the plate) and *S. aureus* (b.1: full plate image b.2: one magnified representative image from the plate).

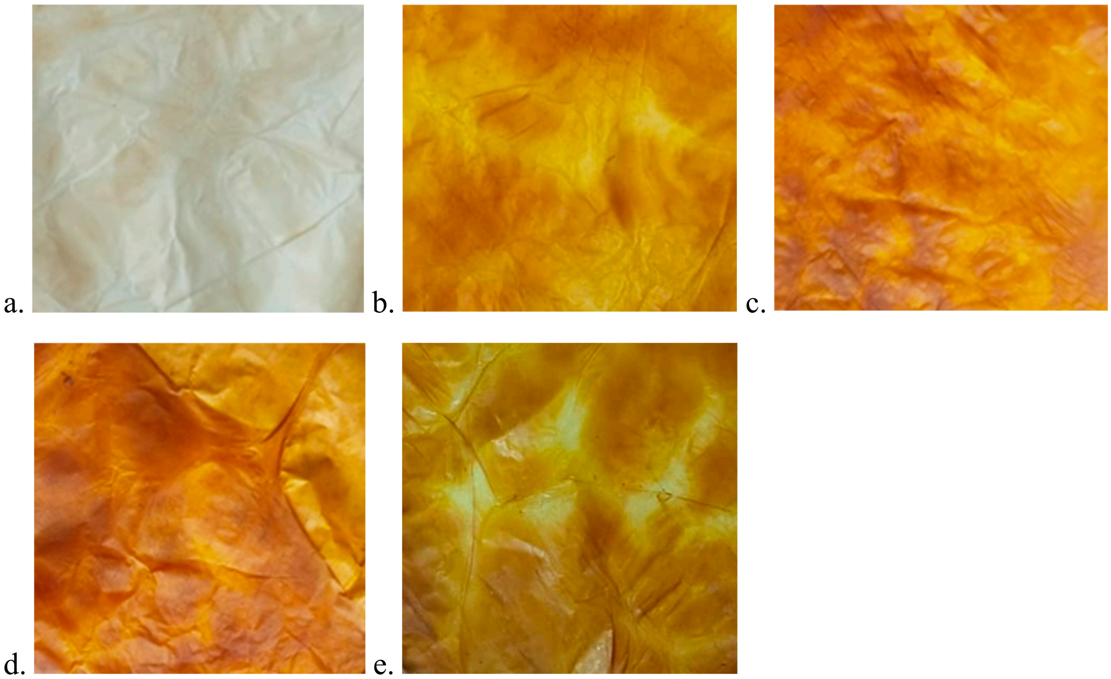

Figure S4. Physical appearance of a. pure bacterial cellulose (BC); b. Ag-BC (without *A. catechu* fruit extract); c. Ag-BC-AC1 (film with 10 mg/ml *A. catechu* fruit extract); d. Ag-BC-AC2 (film with 20 mg/ml *A. catechu* fruit extract); e. Ag-BC-AC3 (film with 30 mg/ml *A. catechu* fruit extract).

Table S1. Physical parameters estimated from XRD analysis

**Sample**	**Crystallinity degree (%)**	**2θ peak cellulose (°)**	**d-spacing (nm)**	**FWHM**	**Scherrer crystallite size (nm)**
Pure bacterial cellulose (BC)	51.26	21.61	0.41	1.54	5.25
Ag-bacterial cellulose without extract	80.93	21.74	0.41	1.42	5.68
Ag-bacterial cellulose with 10 mg/mL extract	81.31	21.61	0.41	1.40	5.77
Ag-bacterial cellulose with 20 mg/mL extract	28.38	21.23	0.41	0.28	6.31
Ag-bacterial cellulose with 30 mg/mL extract			ND		

Table S2. Metabolites compound of *A. catechu* extract

No.	Putative name	Formula	Calc. MW	RT [min]	Area (Max.)	% abd.
1	Arecoline	C8 H13 N O2	155.09442	1.088	6.23E+09	33.5
2	Nicotinyl alcohol	C6 H7 N O	109.05262	0.848	1.92E+09	10.3
3	Guvacoline	C7 H11 N O2	141.07883	1.046	1.07E+09	5.8
4	Caffeine	C8 H10 N4 O2	194.08026	4.164	5.99E+08	3.2
5	Betaine	C5 H11 N O2	117.07898	0.833	5.36E+08	2.9
6	9-Oxo-10(E),12(E)-octadecadienoic acid	C18 H30 O3	294.21924	12.94	5.23E+08	2.8
7	2-[(5Z)-5-tetradecenyl]cyclobutanone	C18 H32 O	264.24511	14.773	4.98E+08	2.7
8	Methyl isonicotinate	C7 H7 N O2	137.04755	0.874	4.34E+08	2.3
9	9,10-Dihydroxystearic acid	C18 H36 O4	316.26081	11.713	3.64E+08	2.0
10	NP-001798	C16 H17 N O2	255.12587	8.336	3.61E+08	1.9
11	(+/-)12(13)-DiHOME	C18 H34 O4	314.24536	11.227	3.28E+08	1.8
12	Stearamide	C18 H37 N O	283.28752	16.704	3.26E+08	1.8
13	Guvacine	C6 H9 N O2	127.06322	0.784	3.15E+08	1.7
14	Cianidanol	C15 H14 O6	290.07875	3.575	2.80E+08	1.5
15	tranexamic acid	C8 H15 N O2	157.10967	1.111	2.71E+08	1.5
16	Phytosphingosine	C18 H39 N O3	317.29247	11.027	2.37E+08	1.3
17	Indane	C9 H10	118.07829	12.855	2.25E+08	1.2
18	Butyryl-L-homoserine lactone	C8 H13 N O3	171.08936	1.215	2.15E+08	1.2
19	Ethyl palmitoleate	C18 H34 O2	282.25591	15.565	2.03E+08	1.1
20	L-α-PALMITIN	C19 H38 O4	330.27658	14.358	1.77E+08	1.0
21	Hexadecanamide	C16 H33 N O	255.25627	14.36	1.44E+08	0.8
22	Protocatechuic acid	C7 H6 O4	154.02654	1.96	1.26E+08	0.7
23	Nootkatone	C15 H22 O	218.16681	12.551	1.23E+08	0.7
24	α-Eleostearic acid	C18 H30 O2	278.22457	13.141	1.21E+08	0.7
25	P-acetylamino phenol	C8 H9 N O2	151.06326	1.029	1.19E+08	0.6
26	Ethyl-2-amino-1-cyclohexene-1-carboxylate	C9 H15 N O2	169.11026	1.48	1.16E+08	0.6
27	1-Stearoylglycerol	C21 H42 O4	358.3078	15.547	1.10E+08	0.6
28	Pyridoxine	C8 H11 N O3	169.07387	3.066	1.04E+08	0.6
29	(+)-Procyanidin B2	C30 H26 O12	578.14171	3.335	1.02E+08	0.6
30	5,7-DIHYDROXY-3′,4′,5′-TRIMETHOXYFLAVANONE	C18 H18 O7	346.10466	10.211	9.88E+07	0.5
31	Penicillic acid	C8 H10 O4	170.05777	3.893	9.74E+07	0.5
32	5-[(9Z)-9-Hexadecenoyloxy]hexadecanoic acid	C32 H60 O4	508.44899	17.786	9.54E+07	0.5
33	nylon cyclic dimer	C12 H22 N2 O2	226.16786	2.778	8.68E+07	0.5
34	Triethyl phosphate	C6 H15 O4 P	182.07075	6.672	8.54E+07	0.5
35	Dihydroartemisinin	C15 H24 O5	284.16237	13.304	7.59E+07	0.4
36	1-Linoleoyl glycerol	C21 H38 O4	354.27659	14.128	6.68E+07	0.4
37	methoxyresorcinol	C7 H8 O3	140.04724	3.065	6.61E+07	0.4
38	2,2,6,6-Tetramethyl-1-piperidinol (TEMPO)	C9 H19 N O	157.14659	9.005	6.48E+07	0.3
39	2-Methoxy-phloroglucinol	C7 H8 O4	156.04209	2.077	5.95E+07	0.3
40	Methyl 5-{(3aS,5R,6R,6aS)-5-hydroxy-6-[(1E,3R)-3-hydroxy-1-octen-1-yl]-1,3a,4,5,6,6a-hexahydro-2-pentalenyl}pentanoate	C22 H36 O4	364.2608	14.502	5.66E+07	0.3
41	(2S)-3-Phenyl-2-({[(3S,4S,5R)-2,3,4-trihydroxy-5-(hydroxymethyl)tetrahydro-2-furanyl]methyl}amino)propanoic acid (non-preferred name)	C15 H21 N O7	327.13124	1.439	5.07E+07	0.3
42	L-Pyroglutamic acid	C5 H7 N O3	129.04254	1.086	4.87E+07	0.3
43	butyrin	C15 H26 O6	302.17296	13.305	4.72E+07	0.3

No.	Putative name	Formula	Calc. MW	RT [min]	Area (Max.)	% abd.
44	1-Linoleoyl-2-Hydroxy-sn-glycero-3-PC	C26 H50 N O7 P	519.33238	12.237	4.64E+07	0.2
45	Caprolactam	C6 H11 N O	113.08415	2.849	4.64E+07	0.2
46	Lauramide	C12 H25 N O	199.1935	13.447	4.40E+07	0.2
47	Nicotinic acid	C6 H5 N O2	123.03195	0.994	4.29E+07	0.2
48	12-Oxo phytodienoic acid	C18 H28 O3	292.20369	10.59	4.02E+07	0.2
49	Dodecylamine	C12 H27 N	185.21414	9.981	3.98E+07	0.2
50	4-Aminophenol	C6 H7 N O	109.05289	3.08	3.92E+07	0.2
51	navenone A	C15 H15 N O	225.11524	10.5	3.79E+07	0.2
52	2-Hydroxychromone	C9 H6 O3	162.03165	3.843	3.77E+07	0.2
53	Adenine	C5 H5 N5	135.0544	0.952	3.77E+07	0.2
54	Oleoyl ethanolamide	C20 H39 N O2	325.29769	14.125	3.57E+07	0.2
55	4-Guanidinobutyric acid	C5 H11 N3 O2	145.08493	0.826	3.43E+07	0.2
56	Guanine	C5 H5 N5 O	151.04939	0.964	3.07E+07	0.2
57	(8E)-2-Amino-8-octadecene-1,3,4-triol	C18 H37 N O3	315.27693	10.612	3.05E+07	0.2
58	Ginkgotoxin	C9 H13 N O3	183.08943	5.194	3.03E+07	0.2
59	(9Z)-9-Cycloheptadecen-1-ol	C17 H32 O	252.24516	11.712	2.89E+07	0.2
60	(2S)-3-Hydroxy-2-(octanoyloxy)propyl henicosanoate	C32 H62 O5	526.4597	17.732	2.74E+07	0.1
61	ACETYL PROLINE	C7 H11 N O3	157.07355	0.85	2.61E+07	0.1
62	Safingol	C18 H39 N O2	301.29769	11.648	2.58E+07	0.1
63	MPTP	C12 H15 N	173.12042	10.23	2.53E+07	0.1
64	(±)-Muscone	C16 H30 O	238.22963	15.196	2.36E+07	0.1
65	Sitostenone	C29 H48 O	412.37029	19.067	2.36E+07	0.1
66	N-(Phenylacetyl)aspartic acid	C12 H13 N O5	251.07924	4.481	2.34E+07	0.1
67	Capsi-amide	C17 H35 N O	269.27187	15.392	2.31E+07	0.1
68	1,3,7-Trihydroxy-6-methoxy-4,5-diisoprenylxanthone	C24 H26 O6	410.17259	14.092	2.25E+07	0.1
69	2-Hydroxy-3-(octanoyloxy)propyl nonadecanoate	C30 H58 O5	498.4283	17.037	2.25E+07	0.1
70	Octadecanamine	C18 H39 N	269.30824	13.288	2.20E+07	0.1
71	Dibenzylamine	C14 H15 N	197.12015	5.812	2.17E+07	0.1
72	acridine-9(10H)-thione	C13 H9 N S	211.04532	11.869	2.16E+07	0.1
73	Bis(2-ethylhexyl) amine	C16 H35 N	241.27692	12.275	2.06E+07	0.1
74	(3β,24R,24′R)-fucosterol epoxide	C29 H48 O2	428.36516	16.615	2.05E+07	0.1
75	Ricinoleic Acid	C18 H34 O3	298.25032	13.821	1.97E+07	0.1
76	2-Phenylpent-4-enal	C11 H12 O	160.08879	10.725	1.95E+07	0.1
77	(2E)-2-Tetradecenal	C14 H26 O	210.19818	14.134	1.92E+07	0.1
78	4-Amino-3-hydroxybenzoic acid	C7 H7 N O3	153.04262	1.87	1.85E+07	0.1
79	Diethyl phosphate	C4 H11 O4 P	154.03942	6.673	1.79E+07	0.1
80	13(S)-HOTrE	C18 H30 O3	294.21922	9.567	1.78E+07	0.1
81	Isorhamnetin	C16 H12 O7	316.05773	6.153	1.69E+07	0.1
82	Erucamide	C22 H43 N O	337.33397	16.56	1.63E+07	0.1
83	1-heptadecanoyl-sn-glycero-3-phosphate	C20 H41 O7 P	424.26106	13.205	1.60E+07	0.1
84	3,5-di-tert-Butyl-4-hydroxybenzaldehyde	C15 H22 O2	234.16193	12.804	1.60E+07	0.1
85	Sedanolide	C12 H18 O2	194.13048	8.989	1.59E+07	0.1
86	5-POHSA	C34 H64 O4	536.48033	18.444	1.57E+07	0.1
87	Adenosine	C10 H13 N5 O4	267.09651	1.071	1.52E+07	0.1
88	(2E,4Z,12E)-1-(1-Piperidinyl)-2,4,12-octadecatrien-1-one	C23 H39 N O	345.30251	16.158	1.42E+07	0.1
89	3-[(1S)-2-Cyclohexen-1-yl]-L-alanine	C9 H15 N O2	169.11026	0.848	1.31E+07	0.1
90	1-(3-Hydroxypropanoyl)-1,3,6,8-tetramethyl-1,2,4a,5,6,7,8,8a-octahydro-2-naphthalenecarboxylic acid	C18 H28 O4	308.19829	12.375	1.31E+07	0.1
91	Betaxolol	C18 H29 N O3	307.21428	8.085	1.15E+07	0.1
92	Bis(2-ethylhexyl) phthalate	C24 H38 O4	390.27648	17.404	1.13E+07	0.1

No.	Putative name	Formula	Calc. MW	RT [min]	Area (Max.)	% abd.
93	1-Palmitoyl-2-hydroxy-sn-glycero-3-PE	C21 H44 N O7 P	453.28511	11.907	1.12E+07	0.1
94	4-Methyl-2,5-dipropylthiazole	C10 H17 N S	183.10777	7.038	1.11E+07	0.1
95	Ethyl myristate	C16 H32 O2	256.24022	16.523	1.08E+07	0.1
96	2-Aminooctadec-4-yne-1,3-diol	C18 H35 N O2	297.26644	10.983	1.06E+07	0.1
97	5α-ergosta-7,22-diene-3β,5-diol	C28 H46 O2	414.34935	16.171	1.03E+07	0.1
98	1-(5,8-Dimethoxy-2,2-dimethyl-2H-chromen-6-yl)ethanone	C15 H18 O4	262.12047	11.017	1.03E+07	0.1
99	Amylbenzene	C11 H16	148.12515	8.989	1.02E+07	0.1
100	3-BHA	C11 H16 O2	180.11496	9.368	1.02E+07	0.1
101	Tridemorph	C19 H39 N O	297.30297	15.864	1.00E+07	0.1
102	Shogaol	C17 H24 O3	276.17241	10.303	9.77E+06	0.1
103	Propofol	C12 H18 O	178.1357	12.877	9.34E+06	0.1
104	16-feruloyloxypalmitic acid	C26 H40 O6	448.2823	14.322	9.32E+06	0.1
105	Monoolein	C21 H40 O4	356.29195	14.734	9.19E+06	<0.1
106	L(-)-2-Amino-3-phenyl-1-propanol	C9 H13 N O	151.09964	10.351	8.52E+06	<0.1
107	1-Tetradecylamine	C14 H31 N	213.24555	10.605	8.24E+06	<0.1
108	Padmatin	C16 H14 O7	318.07329	6.398	8.05E+06	<0.1
109	N-Palmitoyl-L-phenylalanine	C25 H41 N O3	403.30821	15.307	7.07E+06	<0.1
110	6-Hydroxy-5-methyl-4,11-dioxoundecanoic acid	C12 H20 O5	244.13104	6.264	6.65E+06	<0.1

Table S3. Fragmentation profiles of selected compounds from the *A. catechu* extract chromatogram (for analytical evidence)

MS1 fragment	MS2 fragment	Putative Name	Formula	RT [min]
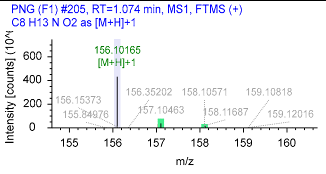	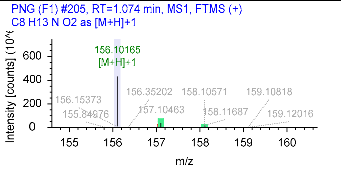	Arecoline 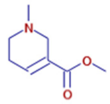	C8 H13 N O2	1.088
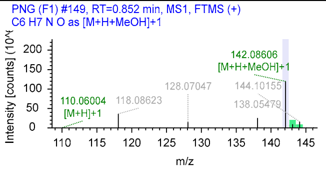	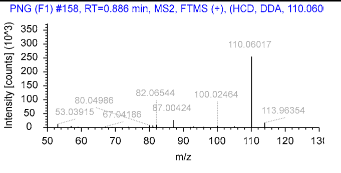	Nicotinyl alcohol 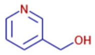	C6 H7 N O	0.848
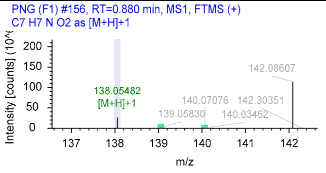	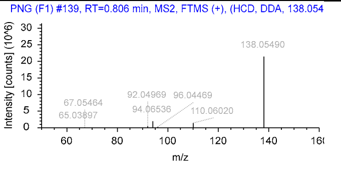	Methyl isonicotinate 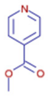	C7 H7 N O2	0.874
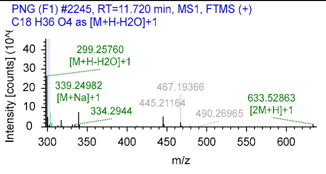	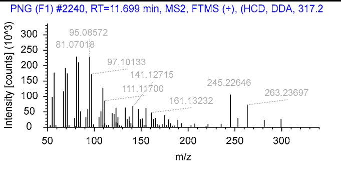	9,10-Dihydroxystearic acid 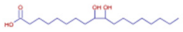	C18 H36 O4	11.713
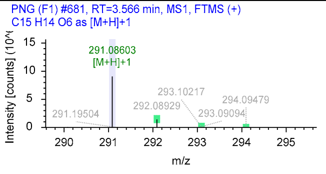	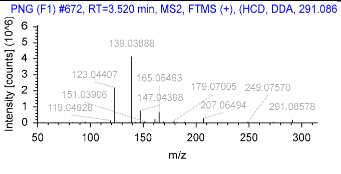	Cianidanol 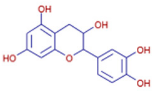	C15 H14 O6	3.575
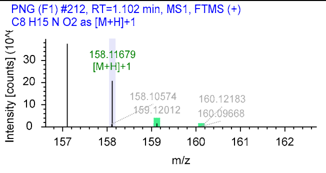	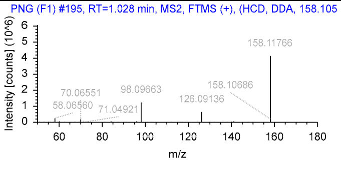	Tranexamic acid 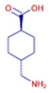	C8 H15 N O2	1.111
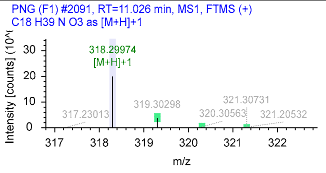	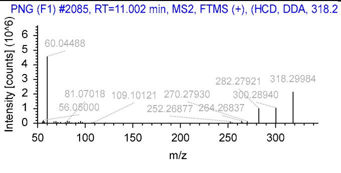	Phytosphingosine 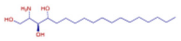	C18 H39 N O3	11.027
